# Reengineering Endogenous Targeting Lipid Nanoparticles (ENDO) for Systemic Delivery of mRNA to Pancreas

**DOI:** 10.1002/adma.202507657

**Published:** 2025-06-12

**Authors:** Ivan Isaac, Luv Patel, Nguyen Tran, Amarnath Singam, DongSoo Yun, Prasun Guha, Seungman Park, Chandrabali Bhattacharya

**Affiliations:** ^1^ Department of Chemistry and Biochemistry University of Nevada Las Vegas Las Vegas NV 89154 USA; ^2^ Nevada Institute of Personalized Medicine University of Nevada Las Vegas Las Vegas NV 89154 USA; ^3^ Department of Mechanical Engineering University of Nevada Las Vegas Las Vegas NV 89154 USA; ^4^ Koch Institute for Integrative Cancer Research Massachusetts Institute of Technology Cambridge MA 02139 USA; ^5^ School of Life Sciences College of Sciences University of Nevada Las Vegas Las Vegas NV 89154 USA; ^6^ Interdisciplinary Biomedical Engineering Program University of Nevada Las Vegas Las Vegas NV 89154 USA

**Keywords:** fifth component LNPs, lipid nanoparticles (LNP), mRNA delivery, pancreas‐targeted delivery, vitamin‐based LNPs

## Abstract

Lipid nanoparticles (LNPs) hold transformative potential for nucleic acid delivery, with applications ranging from clinical use, particularly in COVID‐19 vaccines, to gene therapy and cancer immunotherapy. However, a major limitation lies in their preferential accumulation in the liver following intravenous administration, making most targets hard‐to‐reach. In this study, a novel platform called endogenous targeting lipid nanoparticles (ENDO), containing cholecalciferol (vitamin D3) as a fifth component is reported, that selectively delivers mRNA to the pancreas – a target previously inaccessible through intravenous administration. The top formulation, C‐CholF3, demonstrates an unprecedented 99% pancreas selectivity with robust and sustained protein expression for up to 3 days in a dose‐dependent manner with minimal toxicity that makes it suitable for repeat administration. This organ‐specific delivery is proposed to be mediated by an endogenous targeting mechanism involving the Vitamin D receptor (VDR). C‐CholF3 also enables selective pancreatic delivery of plasmid DNA and circular mRNA, underscoring its versatility and therapeutic potential. Furthermore, C‐CholF3 exhibits pancreas‐specific gene editing in the Ai14 transgenic mouse model, showing high expression of tdTomato in the β cells. These findings highlight its potential for translational applications in protein replacement and CRISPR/Cas9‐mediated gene editing for currently incurable pancreatic diseases, including pancreatic cancer and diabetes.

## Graphical abstract

ENDO lipid nanoparticles containing cholecalciferol as a fifth component demonstrate mRNA delivery to the pancreas with 99% selectivity, including beta cells. This platform shows minimal toxicity and sustained protein expression for up to 3 days in a dose‐dependent manner, offering a novel approach to pancreas‐specific mRNA delivery and gene therapy that is suitable for repeat administration with clinical relevance.

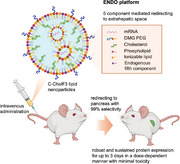

## 1 Introduction

Lipid nanoparticles (LNPs) have revolutionized the field of nucleic acid delivery and have been extensively explored in gene therapy, protein replacement therapies, and immunotherapies for various diseases.^[^ ^1^, ^2^, ^3^ ^]^ Their clinical applications were notably demonstrated by their widespread use in delivering the SARS‐CoV‐2 vaccine during the COVID‐19 pandemic.^[^ ^4^, ^5^, ^6^, ^7^ ^]^ Despite their promise, targeting beyond the liver remains a significant challenge with the traditional LNPs, which are made up of four key components—ionizable lipid, phospholipid, cholesterol, and polyethylene glycol (PEG) lipid.^[^ ^5^, ^8^, ^9^ ^]^ The accumulation of LNPs in the liver primarily occurs because they associate with Apolipoprotein E (ApoE) in the blood, which is crucial for cholesterol transport and low‐density lipoprotein receptor (LDLR)‐mediated endocytosis.^[^ ^10^, ^11^, ^12^, ^13^ ^]^ The mode of administration also plays a critical role in redirecting LNPs to extrahepatic sites. Previously, intraperitoneal administration effectively redirected nanoparticles to the pancreas with robust and specific protein expression via macrophage‐mediated gene transfer.^[^ ^14^ ^]^ Another promising approach for redirecting LNPs to extrahepatic sites is incorporating a fifth component in the traditional LNP formulation. This strategy has been effective in achieving lung‐ and spleen‐specific mRNA expression through interactions with plasma proteins, such as vitronectin (Vtn) and β2 glycoprotein 1 (β2‐GPI), using SORT LNPs containing a fifth component cationic or anionic lipid.^[^ ^15^, ^16^, ^17^, ^18^, ^19^ ^]^ Furthermore, the addition of an adjuvant lipidoid containing a Toll‐like receptor (TLR) 7/8 agonist as a fifth component also successfully demonstrated transfection in the lymph nodes.^[^ ^20^ ^]^ This illustrates the potential of LNPs to target other hard‐to‐reach tissues with formulation design, overcoming the inherent limitations of classical four‐component LNPs.

**Figure 1:**
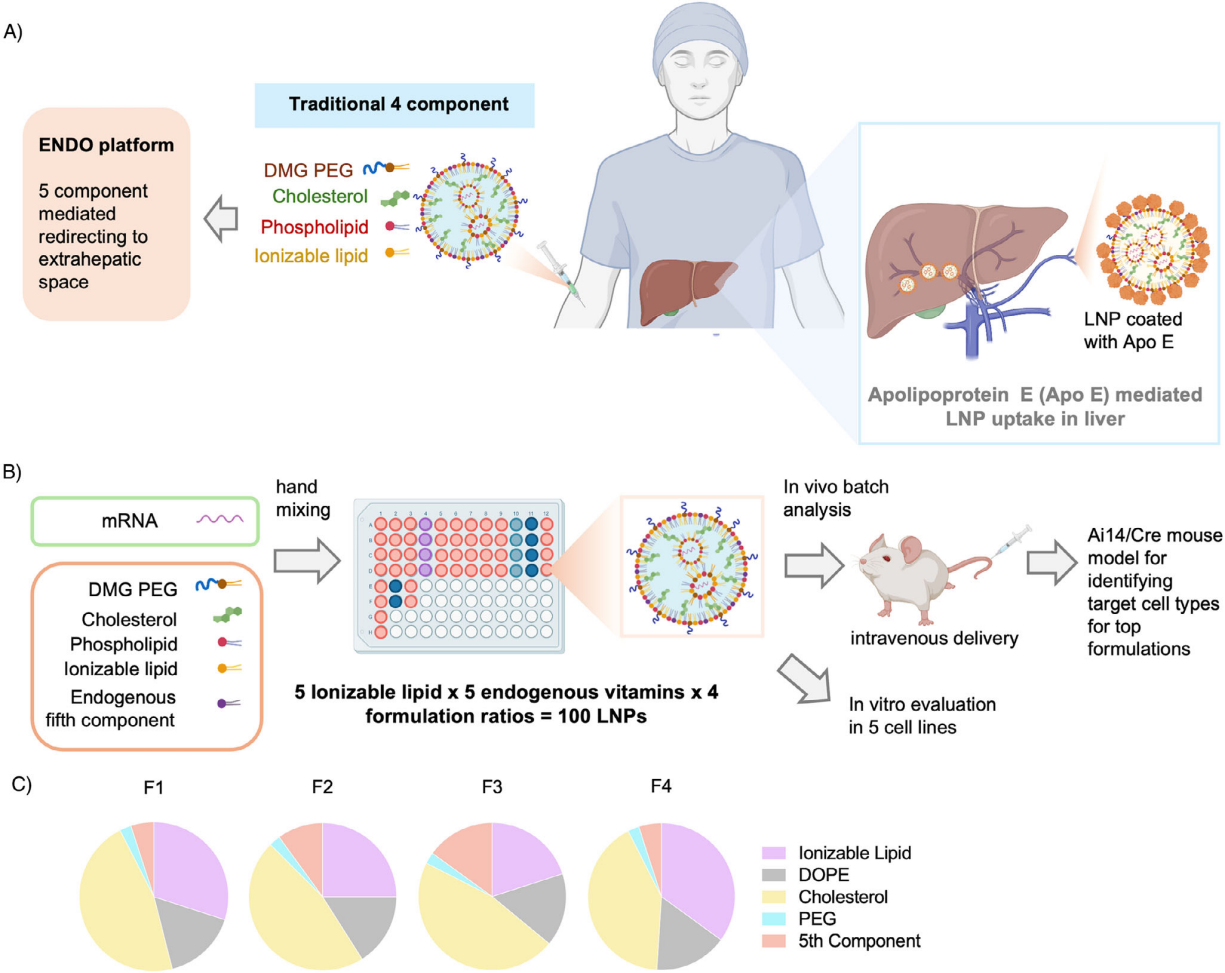
Overview of the fifth component ENDO LNP formulation and screening platform. A) Schematic representation illustrating the current challenges in developing a robust extrahepatic delivery system. B) Schematic showing ENDO LNP formulation and screening platform. C) Pie charts depicting the different formulation ratios for ionizable lipid, phospholipid, cholesterol, PEG lipid, and endogenous vitamin fifth component.

Here, we report a novel platform called endogenous targeting lipid nanoparticles (ENDO), which incorporates endogenous ligands such as vitamins as the fifth component in the LNP formulation, fine‐tuning their organ tropism and enhancing targeted delivery (**Figure** **1**A). We chose endogenous vitamins because of their distinct chemical structures and unique biochemical functions, which make them ideal candidates for the fifth component platform.^[^ ^21^, ^22^, ^23^ ^]^ Structurally, vitamins can be broadly divided into water‐soluble groups, such as the vitamin B complex, and fat‐soluble groups, which include vitamins A, D, E, and K.^[^ ^24^, ^25^ ^]^ The fat‐soluble vitamins are particularly relevant to the LNP formulations due to their hydrophobic tails, which can integrate seamlessly into the lipid bilayer.^[^ ^26^, ^27^ ^]^ Additionally, ionizable lipids containing vitamin C have been shown to improve transfection efficiency and exhibit anti‐microbial properties against sepsis.^[^ ^28^ ^]^ In this study, we screened a library of 100 LNPs formulated with various combinations of fifth‐component endogenous vitamins, different ionizable lipids, and distinct formulation ratios to assess their potential for tissue‐specific delivery (Figure 1B). The resulting ENDO LNPs were tested in five different cell lines representing major cell types in the body and demonstrated varying levels of transfection across these cell types. We further investigated the ability of these LNPs to deliver mRNA in vivo using batch analysis.

This screening led to the identification of two formulations containing cholecalciferol as a fifth component, which demonstrated selective delivery of mRNA to the pancreas with high efficacy through intravenous administration–C‐CholF2 and C‐CholF3. Among these formulations, C‐CholF3 exhibited higher protein expression than C‐CholF2, achieving over 99% selectivity and sustained expression in the pancreas for up to 3 days in a dose‐dependent manner. We propose that the pancreas tropism of C‐CholF3 arises from an endogenous targeting mechanism involving the Vitamin D receptor (VDR), based on loss of pancreatic protein expression following VDR blockade using MeTC7 antagonist. In addition to mRNA, C‐CholF3 facilitates selective pancreatic delivery of other nucleic acid cargos, including both plasmid DNA and circular mRNA, establishing a broadly compatible nucleic acid delivery platform for diverse therapeutic applications. To our knowledge, no existing material or LNP formulation has demonstrated the capability to deliver mRNA specifically to the pancreas via intravenous administration. The addition of biocompatible vitamins also resulted in reduced toxicity, improved safety profiles, and enhanced tolerance. Furthermore, we assessed the gene editing ability of C‐CholF3 LNP in the Ai14 mouse model and observed tissue‐specific gene editing in the pancreas, with efficient expression of tdTomato in β cells. In summary, the ENDO platform highlights the importance of incorporating endogenous vitamins as the fifth component of LNPs for targeted delivery to specific tissues. Furthermore, it broadens the potential for targeting hard‐to‐reach extrahepatic organs, such as the pancreas, and provides solutions for protein replacement and CRISPR/Cas9‐mediated gene editing to treat currently incurable pancreatic diseases.

## 2 Results and Discussion

Traditional LNPs for mRNA delivery consist of four key components, each tailored to establish a stable lipid bilayer structure. These lipids include 1) ionizable lipids for complexing with mRNA with charge‐altering properties, facilitating the release of mRNA cargo in the acidic endosomal environment; 2) helper lipids for bilayer structure reinforcement; 3) cholesterol for increasing structural integrity; and 4) PEG lipids for extending circulation in the bloodstream.^[^ ^29^, ^30^, ^31^, ^32^ ^]^ To enhance organ‐specific delivery of LNPs, we added endogenous vitamins as a fifth component in the formulation. The vitamins A, B2, D3, E, and K1 were chosen for their unique biological functions, which can complement the LNP core in different ways. Vitamin A (retinol) is well‐known for its role in cell differentiation and immune modulation.^[^ ^33^, ^34^ ^]^ Vitamin B2 (riboflavin), although water‐soluble, was included because it plays a crucial role in cellular metabolism and energy production.^[^ ^35^, ^36^ ^]^ Vitamin D3 (cholecalciferol) is essential for calcium homeostasis and immune function, specifically targeting tissues that express the VDR.^[^ ^37^ ^]^ This vitamin is necessary for maintaining β‐cell function, supporting insulin release, reducing β‐cell apoptosis, and promoting overall cell health.^[^ ^38^ ^]^ Vitamin E (tocopherol) possesses significant antioxidant properties, and vitamin K1 (phylloquinone) is crucial for blood coagulation and bone health.^[^ ^39^, ^40^ ^]^

To further diversify our ENDO LNP design, we developed a comprehensive formulation library using previously studied ionizable lipids (ILs), including benchmark SM‐102, DLin‐MC3‐DMA (MC3), and C12‐200, which are known to traffic to the liver.^[^ ^41^, ^42^, ^43^ ^]^ Additionally, we used THP1, which our research group previously established as an effective mRNA delivery system.^[^ ^44^ ^]^ 306Oi10 was included due to its previously demonstrated high transfection efficiency in numerous preclinical studies.^[^ ^45^ ^]^ For an efficient fifth component formulation development, it is crucial to partially replace the ionizable lipid without affecting encapsulation or endosomal escape. We started our study by using a benchmark formulation containing ionizable lipid, helper lipid, cholesterol, and DMG‐PEG 2000 at a 35:16:46.5:2.5 molar ratio, which was then modified by partially replacing the ionizable lipid or cholesterol. The four different formulation ratios were chosen with an increasing vitamin substitution (5–15 molar ratios) to systematically investigate the effect of vitamin molar ratios on the performances of these LNPs. The detailed formulation ratios are shown in Figure 1C and Table S1 (Supporting Information). Designing the formulation ratio is as significant as selecting the suitable fifth component in this study as it impacts the physicochemical parameters of LNP, including particle size, encapsulation efficiency (EE%), and surface charge, which are important for efficient mRNA delivery. These interactions can change significantly with minor alterations in the lipid‐to‐vitamin ratio, affecting the stability of particles in circulation and their abilityto escape endosomes. We formulated a library of 100 different LNP formulations by combining five different ILs, five vitamins, and four different formulation ratios (5 ILs × 5 Vitamins × 4 Formulation = 100 LNPs) in a 96‐well plate format using hand mixing. We used firefly luciferase (FLuc) mRNA as our reporter because its non‐secretory nature allows direct imaging and quantification of transfection efficiency. The particle size and PDI of each LNP formulation were measured using dynamic light scattering (DLS), as shown in Figures S1 and S2 (Supporting Information). The results indicate that the average diameter of most LNPs was within a window of 60 to 140 nm and PDIs smaller than 0.2, indicating a relatively narrow size distribution. The uniformity ensures efficient cellular uptake and successful endosomal escape, demonstrating that ENDO LNPs were structurally similar to conventional LNPs.

**Figure 2:**
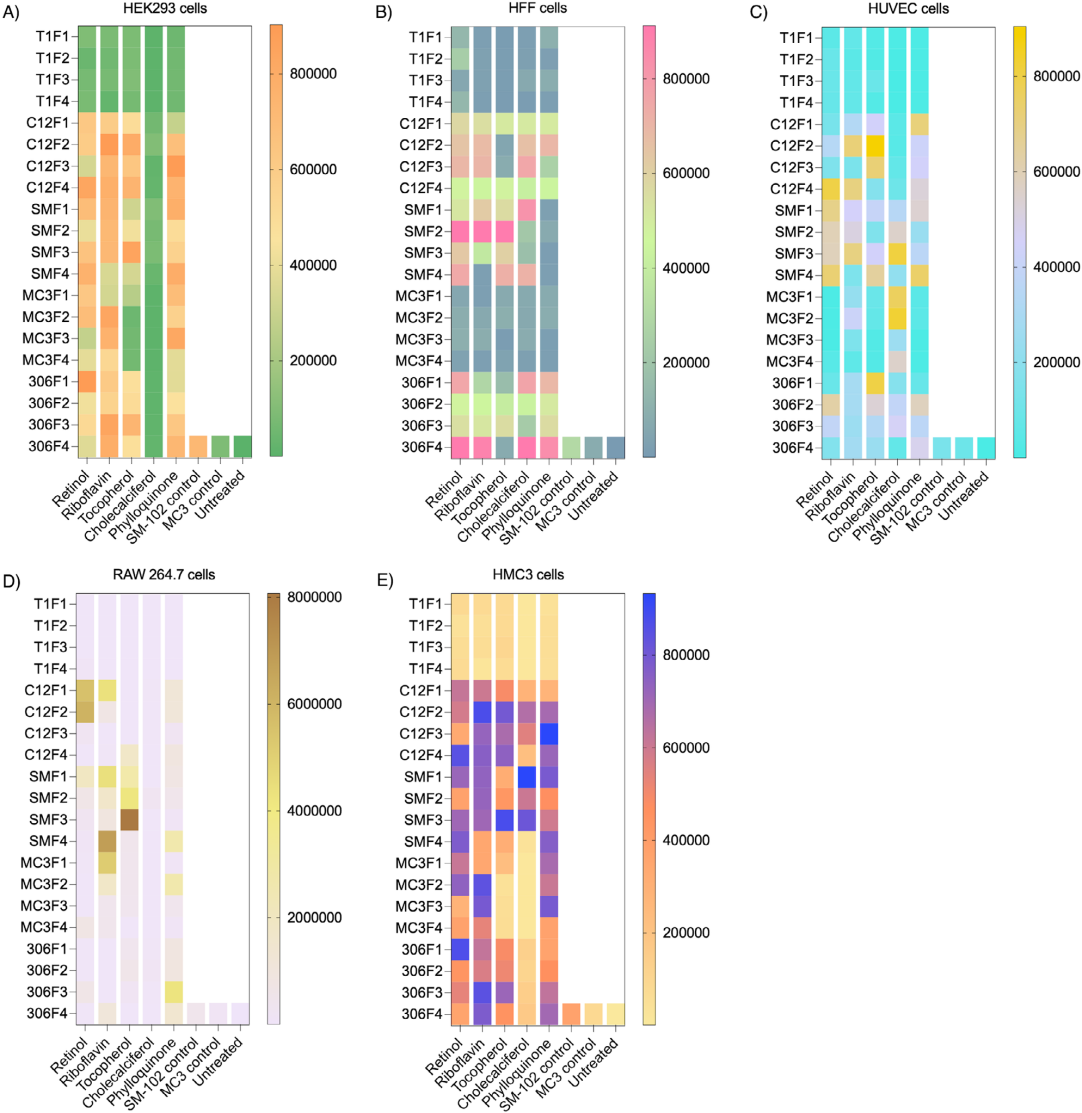
In Vitro screening of ENDO LNPs. A) Heat map of in vitro delivery of luciferase mRNA with ENDO LNPs in HEK293 cells (125 ng mRNA per well, 96 well plate, *n* = 3). Luminescence intensity was quantified 24 h after treatment with LNPs. B) Heat map of in vitro delivery of luciferase mRNA with ENDO LNPs in HFF cells (125 ng mRNA per well, 96 well plate, *n* = 3). Luminescence intensity was quantified 24 h after treatment with LNPs. C) Heat map of in vitro delivery of luciferase mRNA with ENDO LNPs in HUVEC cells (125 ng mRNA per well, 96 well plate, *n* = 3). Luminescence intensity was quantified 24 h after treatment with LNPs. D) Heat map of in vitro delivery of luciferase mRNA with ENDO LNPs in RAW 264.7 cells (125 ng mRNA per well, 96 well plate, *n* = 3). Luminescence intensity was quantified 24 h after treatment with LNPs. E) Heat map of in vitro delivery of luciferase mRNA with ENDO LNPs in HMC3 cells (125 ng mRNA per well, 96 well plate, *n* = 3). Luminescence intensity was quantified 24 h after treatment with LNPs. Abbreviation used: T1, THP1; C12, C12‐200; SM, SM‐102; 306, 306Oi10; F1, Formulation1; F2, Formulation2; F3, Formulation3; F4, Formulation4.

We assessed the in vitro transfection efficiencies of all the LNPs in various cell lines, including human embryonic kidney cells (HEK293), human foreskin fibroblasts (HFF), human umbilical vein endothelial cells (HUVEC), mouse macrophage cells (RAW264.7), and human microglia cells (HMC3). These cell types represent a diverse range of tissues, including the kidney, skin, vascular endothelium, immune system, and brain. A comprehensive analysis of the transfection data revealed several key trends, indicating a significant improvement in transfection by ENDO LNPs compared with control MC3 and SM‐102. Notably, LNPs incorporating retinol, riboflavin, and phylloquinone as the fifth component exhibited significantly higher transfection efficiency in HEK293 cells compared to other ENDO LNPs and the control group (**Figure** **2**A). In contrast, LNPs containing cholecalciferol as the fifth component and those with THP1 as the ionizable lipid showed reduced transfection efficiency in HEK293 cells. In HFF cells, SM‐102 LNPs incorporating retinol, riboflavin, and tocopherol as a fifth component, as well as 306Oi10 LNPs with retinol, riboflavin, and cholecalciferol, achieved transfection levels several‐fold higher than control LNPs and other ENDO LNPs (Figure 2B). Furthermore, formulations containing MC3 and THP1 as ionizable lipids exhibited lower transfection efficiency in HFF cells. In HUVEC cells, which form the inner lining of blood vessels, C12‐200 LNPs with retinol and tocopherol, 306Oi10‐based LNPs with tocopherol, and SM‐102 and MC3 LNPs containing cholecalciferol demonstrated significantly higher transfection than control LNPs and other ENDO LNPs (Figure 2C). THP1 LNPs showed poor transfection in HUVEC cells. RAW264.7 cells, which resemble macrophages, displayed significantly higher transfection with C12‐200‐based LNPs containing retinol. Notably, SM‐102‐based LNPs incorporating tocopherol achieved the highest transfection levels in these cells (Figure 2D), likely due to enhanced cellular uptake and intracellular processing of the LNPs. Other LNP formulations showed limited transfection in RAW264.7 cells. In HMC3 microglial cells, C12‐200 LNPs containing retinol, riboflavin, and phylloquinone exhibited several‐fold higher transfection efficiency than controls (Figure 2E). Similarly, SM‐102 LNPs containing tocopherol and cholecalciferol, as well as MC3 LNPs with riboflavin and 306Oi10 ionizable lipids combined with retinol and riboflavin, demonstrated enhanced transfection. However, LNPs formulated with THP1 as the ionizable lipid showed low transfection in HMC3 cells. Further, all the traditional four‐component formulations of C12‐200, SM‐102, 306Oi10, THP1, and MC3 were tested across all the cell lines, and none exhibited superior transfection efficiency compared to the highlighted formulations in our study (Figure S3, Supporting Information). Hence, our findings highlight that incorporating the vitamins as a fifth component into the ENDO LNPs can greatly enhance transfection efficacy compared with traditional four‐component LNPs, providing a rationale for tissue‐specific delivery.

**Figure 3:**
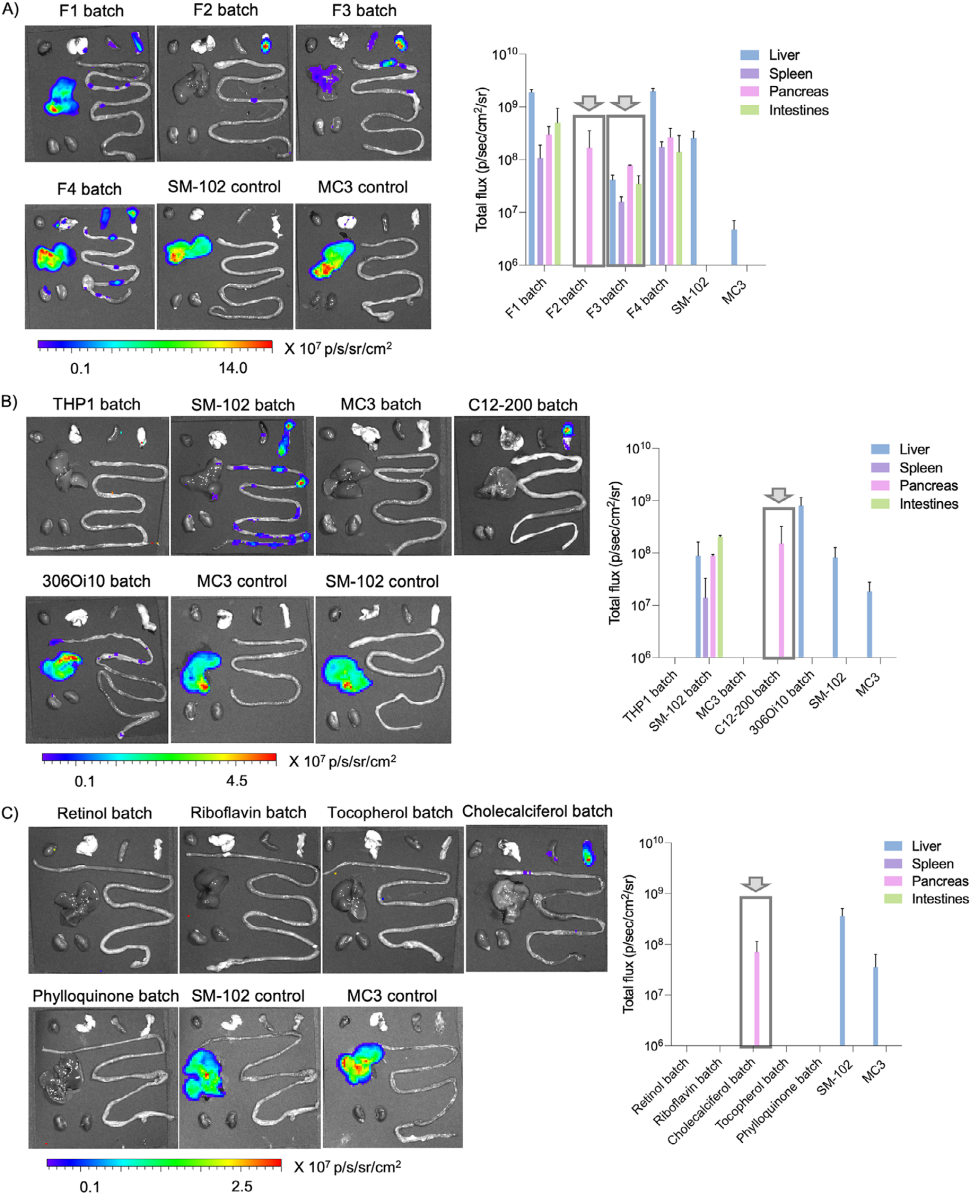
In Vivo screening of ENDO LNPs using batch analysis. A) IVIS images at 24 h post‐injection and graphical representation of total flux of FLuc mRNA ENDO LNP pool based on formulations (F1, F2, F3, and F4). SM‐102 and MC3 were used as a control. C57BL/6 mice were injected intravenously with 0.5 mg kg^−1^ of pooled ENDO LNPs. (*n* = 2 biologically independent mice, ± SD). Organs are arranged left to right as: heart, lung, spleen, pancreas, liver, intestines, and kidneys. B) IVIS images at 24 h post‐injection and graphical representation of total flux of FLuc mRNA ENDO LNP pool based on ionizable lipids. Pancreas targeting F2 and F3 LNPs were pooled and batched based on the respective ionizable lipids (THP1, C12‐200, SM‐102, MC3, 306Oi10) and injected intravenously. SM‐102 and MC3 were used as a control. C57BL/6 mice were injected intravenously with 0.5 mg kg^−1^ of pooled ENDO LNPs. (*n* = 2 biologically independent mice, ± SD). Organs are arranged left to right as: heart, lung, spleen, pancreas, liver, intestines, and kidneys. C) IVIS images at 24 h post‐injection and graphical representation of total flux of FLuc mRNA ENDO LNP pool based on endogenous vitamins. Selective pancreas targeting C12‐200 ENDO LNPs were then batched based on vitamins A (retinol), B2 (riboflavin), D3 (cholecalciferol), E (tocopherol), and K1 (phylloquinone). SM‐102 and MC3 were used as a control.  C57BL/6 mice were injected intravenously with 0.5 mg kg^−1^ of pooled ENDO LNPs. (*n* = 2 biologically independent mice, ± SD). Organs are arranged left to right as: heart, lung, spleen, pancreas, liver, intestines, and kidneys.

Next, we evaluated the in vivo delivery efficacy of the ENDO LNPs using comprehensive batch analysis. This approach accelerated our screening process significantly and resulted in a remarkable reduction in the number of animals, as well as time and cost. In this study, we categorized 100 LNPs into four groups based on their formulation ratios (F1, F2, F3, and F4). Each group, containing 25 formulations corresponding to its respective formulation ratio, was administered to C57BL/6 mice via intravenous injection at a dosage of 0.5 mg kg^−1^, and protein expression was assessed in major organs using an IVIS imaging system. Formulations F1 and F4 primarily showed transfection in the liver and, with additional expression in the spleen, intestines, and pancreas. Notably, formulations F2 and F3 demonstrated significant redirection to extrahepatic space, with F2 showing selective protein expression in the pancreas and minimal expression in other organs, including the spleen and liver (**Figure** **3**A). Extrahepatic formulations, F2 and F3, were then batched based on ionizable lipids (THP1, C12‐200, SM‐102, MC3, and 306Oi10) in five groups, allowing for a total administration of 10 LNPs per injection at the same dose. Remarkably, C12‐200 batch showed selective transfection in the pancreas, whereas the other ILs resulted in broader expression across various organs (Figure 3B). This highlights the significance of selecting the appropriate IL to achieve targeted mRNA delivery. Further, C12‐200 batch was grouped based on different vitamins (retinol, riboflavin, cholecalciferol, tocopherol, and phylloquinone). Among these, cholecalciferol exhibited selective mRNA transfection and protein expression in the pancreas, highlighting the potential of ENDO LNPs for more precise mRNA delivery (Figure 3C). Throughout each phase of the screening process, SM‐102 and MC3 were used as control LNPs with major accumulation in the liver.

**Figure 4:**
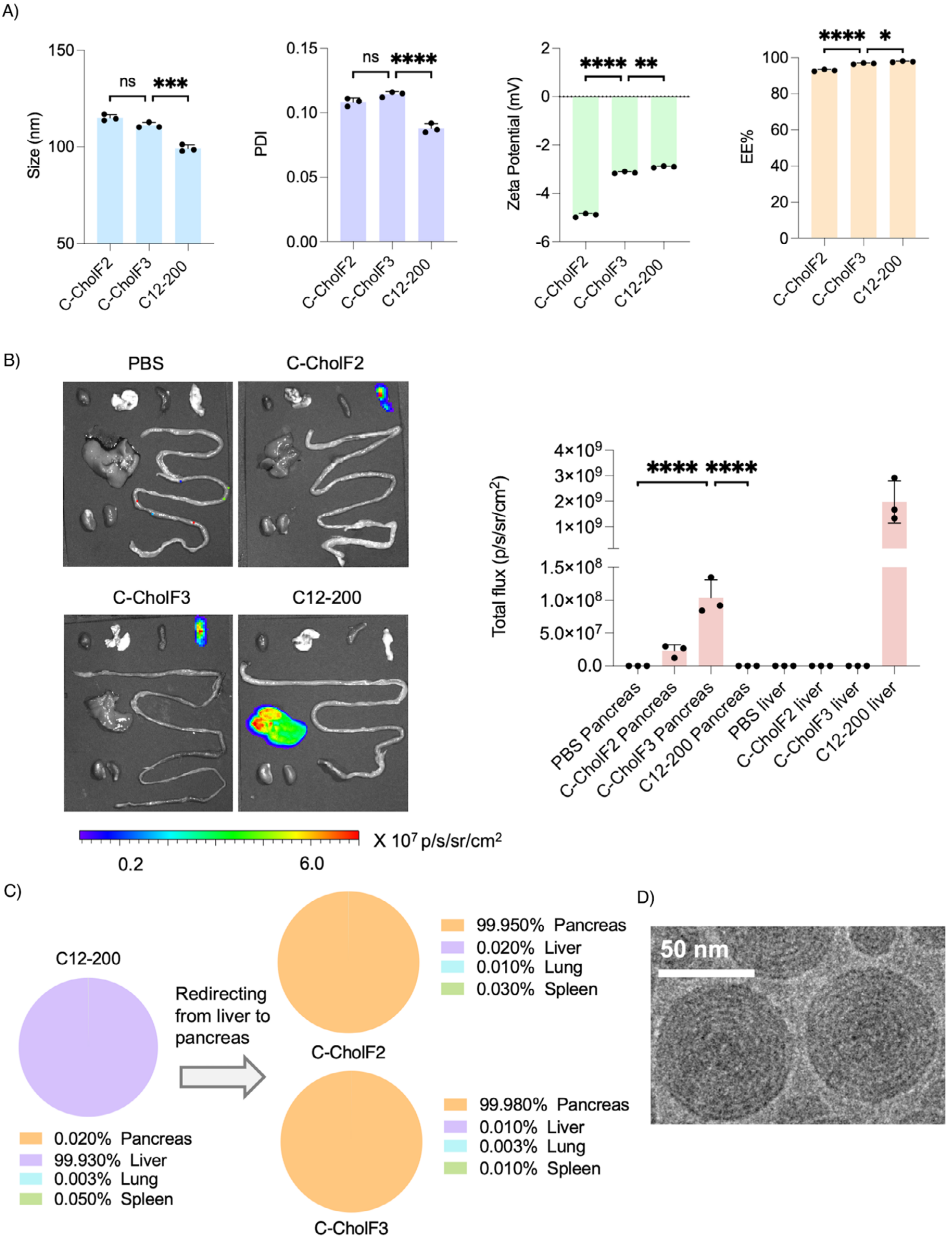
Validation of top ENDO LNPs, C‐CholF2 and C‐CholF3. A) Physiochemical properties such as size, PDI, *ζ*‐potential measurements, and mRNA encapsulation efficiency of C‐CholF2 and C‐CholF3 along with control (C12‐200). (*n* = 3, ± SD, ^*^ *p* < 0.05, ^**^ *p* < 0.01, ^***^ *p* < 0.001, ^****^ *p* < 0.0001. NS, not significant, one‐way ANOVA with Bonferroni post‐hoc analysis). B) Representative IVIS images at 24 h post‐injection and graphical representation of total flux of C‐CholF2 and C‐CholF3 ENDO LNPs injected intravenously at a dose of 0.5 mg kg^−1^. PBS and C12‐200 were also injected as a control (*n* = 3 biologically independent mice, ± SD, ^*^ *p* < 0.05, ^**^ *p* < 0.01, ^***^ *p* < 0.001, ^****^ *p* < 0.0001. NS, not significant, one‐way ANOVA with Bonferroni post‐hoc analysis). Organs are arranged left to right as: heart, lung, spleen, pancreas, liver, intestines, and kidneys. C) Pie charts illustrating the percentage of protein expression occurring per organ after intravenous injection of C12‐200 (four component traditional components) and redirection to the pancreas for C‐CholF2 and C‐CholF3 LNPs (*n* = 3 biologically independent mice). D) Representative cryo‐EM image of C‐CholF3 LNPs. Scale bar, 50 nm.

To further validate our findings from the batch screening, we formulated C12‐200 cholecalciferol F2 (C‐CholF2) and C12‐200 cholecalciferol F3 (C‐CholF3) LNPs using a microfluidic device. Previous studies have shown that LNPs formulated via hand mixing and microfluidic mixing exhibit no significant differences in transfection, both in vitro and in vivo.^[^ ^8^, ^17^, ^44^ ^]^ Microfluidic mixing allows improved reproducibility by accurately mixing aqueous and ethanol phases, resulting in highly reproducible LNPs with desired physicochemical properties essential for clinical translation and large‐scale production. The measurement of particle size and EE% of these LNPs indicated that both C‐CholF2 and CholF3 had similar sizes and notably high EE%, demonstrating effective mRNA encapsulation (**Figure** **4**A). To validate the efficacy of these ENDO formulations in the pancreas, we intravenously injected each LNP at 0.5 mg kg^−1^ in C57BL/6 mice, along with the C12‐200 control. The results demonstrated that both C‐CholF3 and CholF2 exhibited selective expression in the pancreas, with an average total flux of 1.04 × 10⁸ for C‐CholF3 compared to 2.29 × 10⁷ for CholF2, indicating a 4.5‐fold higher efficacy (Figure 4B; Figure S4, Supporting Information). Thus, C‐CholF3 can selectively target the pancreas with significantly higher efficiency, demonstrating a complete redirection of liver‐targeting C12‐200 LNPs with cholecalciferol (Figure 4C). To further evaluate its versatility, we assessed the efficiency of C‐CholF3 via the intraperitoneal route. Although the average total flux was reduced to 5.94 × 10⁶ (≈12% of the intravenous signal), the selectivity for the pancreas remained above 99% (Figure S5, Supporting Information). These findings highlight that the pancreas‐targeting capability of C‐CholF3 is preserved even when delivered intraperitoneally, broadening its potential for alternative delivery applications. The cryogenic electron microscopy (cryo‐EM) image of C‐CholF3 ENDO LNPs showed a spherical shape with a multi‐lamellar shell surrounding an amorphous core (Figure 4D; Figure S6, Supporting Information). To assess the dose‐dependency of C‐CholF3 and understand whether the protein expression in the pancreas could be regulated, we intravenously administered 0.25, 0.5, and 1 mg kg^−1^ of C‐CholF3 LNPs to C57BL/6 mice and observed a distinguishable dose‐dependent improvement in transfection efficiency (Figure S7, Supporting Information). This highlights the importance of optimizing dosing to maximize the efficiency of LNP‐mediated mRNA delivery along with balancing safety at higher doses. These findings will be crucial in determining the therapeutic window and the dosing strategies of future clinical applications.

**Figure 5:**
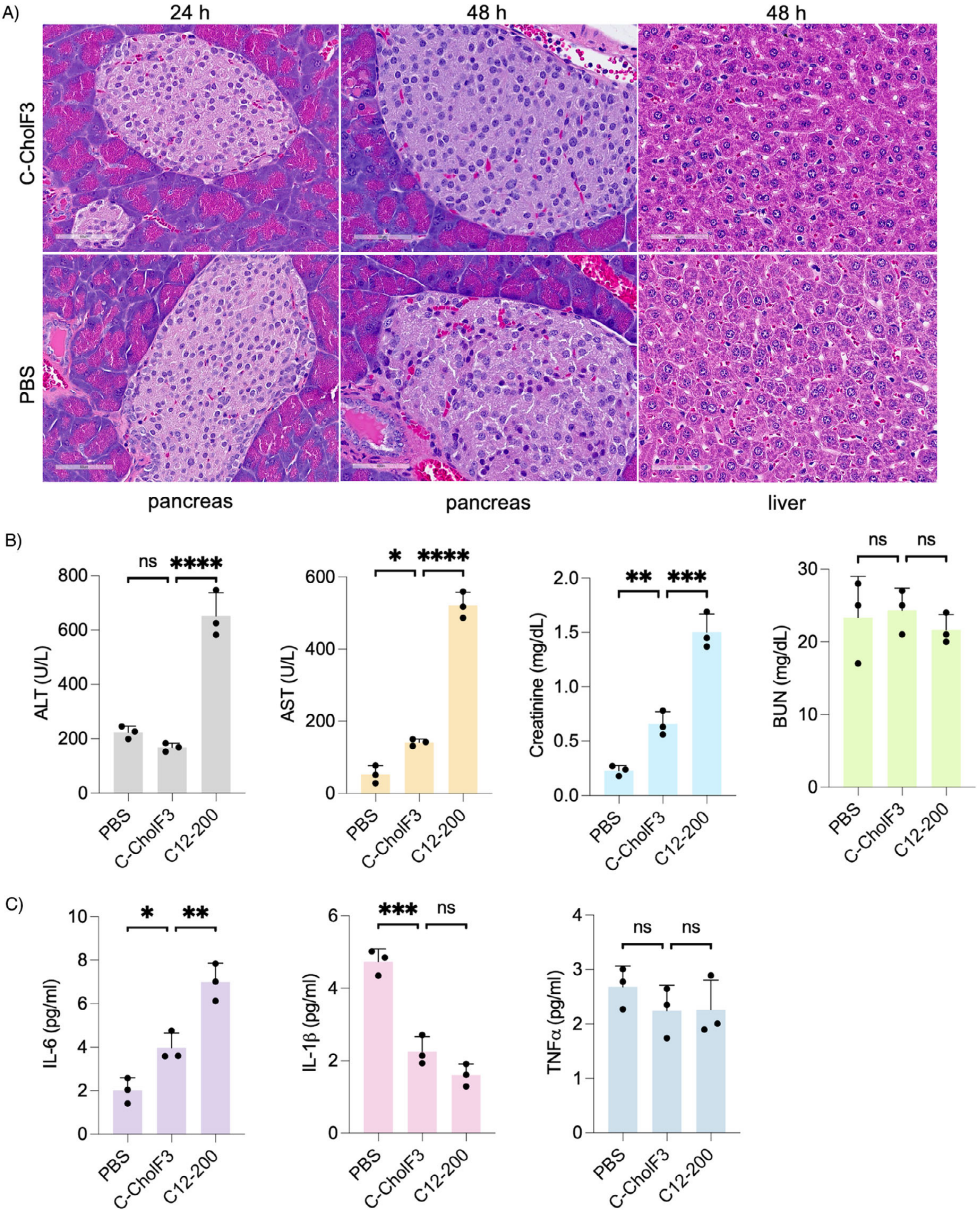
Toxicity and safety evaluation of C‐CholF3 ENDO LNPs. A) Representative histology images of the pancreas (24 and 48 h post‐treatment) and liver sections 48 h post‐treatment with C‐CholF3 encapsulating FLuc mRNA, and PBS (control) via intravenous injection in C57BL/6 mice at a dose of 0.5 mg kg^−1^. Hematoxylin and Eosin (H&E) staining was performed, with images taken at 40× magnification. Scale bars: 60 µm (*n* = 3 biologically independent mice). B) Serum levels of liver enzymes, alanine aminotransferase (ALT) and aspartate aminotransferase (AST), renal parameters, blood urea nitrogen (BUN), and creatinine 24 h after intravenous administration with PBS, C‐CholF3 and C12‐200 LNPs at a dose of 0.5 mg kg^−1^ (*n* = 3, ± SD, ^*^ *p* < 0.05, ^**^ *p* < 0.01, ^***^ *p* < 0.001, ^****^ *p* < 0.0001; NS, not significant, one‐way ANOVA with Bonferroni post hoc analysis). C) Levels of IL‐6, IL‐1β, and TNFα in mice intravenously treated with C‐CholF3 and C12‐200 LNPs at a dose of 0.5 mg kg^−1^ (*n* = 3 biologically independent mice, ± SD, ^*^ *p* < 0.05, ^**^ *p* < 0.01, ^***^ *p* < 0.001; NS indicates no significance, one‐way ANOVA with Bonferroni post‐hoc analysis). PBS‐injected mice were kept as the control group.

Next, we investigated the in vivo biocompatibility of C‐CholF3. H&E staining, both after 24 and 48 h, showed no morphological damage or inflammatory response in pancreatic tissues treated with C‐CholF3 compared to the PBS‐treated control (**Figure** **5**A; Figures S8 and S9, Supporting Information). The islet cells remained intact, and there was no evidence of necrosis or degeneration. Additionally, the liver tissues were examined with H&E staining after 48 h and showed no significant change compared to the control. These findings highlight the safety and tolerability of C‐CholF3 and its potential for future clinical applications. In addition to histopathological examination, the overall safety profile of C‐CholF3 LNPs was studied by continuously monitoring body weight and hematological parameters. Over 21 days, no significant decrease in body weight was observed in mice (Figure S10, Supporting Information). The levels of liver enzymes such as ALT, AST, and alkaline phosphatase were within the normal range, with no renal toxicity observed based on the levels of BUN and CREA (Figure 5B; Figure S11, Supporting Information). It is known that different LNPs can elicit varied cytokine responses depending on factors such as lipid composition, nanoparticle size, and surface properties, leading to differential activation of immune pathways.^[^ ^46^, ^47^, ^48^ ^]^ Proinflammatory cytokines, such as IL‐1β, IL‐6, and TNF‐α, were measured 24 h after injection and found to be comparable to PBS control, further confirming the low immunogenicity of the LNP (Figure 5C). Other immune biomarkers, including GM‐CSF, IFNγ, IL‐2, IL‐4, IL‐10, IL‐12p70, and MCP‐1, also showed comparable levels to the control, indicating minimal systemic inflammatory response that is ideal for repeat dosing of LNPs (Figure S12, Supporting Information). These findings highlight that C‐CholF3 ENDO LNPs exhibit significantly higher biocompatibility and reduced toxicity compared to traditional C12‐200 LNPs. This is likely due to the replacement of a substantial portion of C12‐200 ionizable lipid in the standard four‐component formulation with endogenous cholecalciferol, a naturally occurring compound, thereby improving safety and biocompatibility.

To ensure robustness and the stability of C‐CholF3 LNPs for clinical applications, we stored them at both 4 and −20 °C for various durations (1, 3, 7, and 21 days) and evaluated changes in their physiochemical properties and mRNA delivery efficacy. Throughout all storage conditions, the LNPs exhibited minimal changes in physiochemical properties, with parameters such as particle size, polydispersity index (PDI), ζ−potential, and EE% being consistent. Notably, the LNPs demonstrated robust FLuc expression even after 21 days of storage without any cryoprotectants, indicating exceptional stability (Figure S13, Supporting Information). This suggests that C‐CholF3 LNPs can maintain their mRNA delivery potential over extended storage periods and can be adopted for long‐term storage and transport, making them suitable for clinical and commercial use. We also assessed the in vivo stability and kinetics of C‐CholF3 LNPs to understand and determine their potential for extended‐release. For this, we administered the nanoparticles intravenously into C57BL/6 mice and monitored the bioluminescence signal over 72 h. We observed that the bioluminescence intensity remained robust for up to 72 h post‐injection, indicating prolonged stability and expression of the delivered mRNA (Figure S14, Supporting Information). The higher stability of C‐CholF3 LNPs could be advantageous for applications requiring prolonged gene expression in vivo.

Organ tropism has long been understood to be influenced by the apparent pKa of LNPs, with lower pKa values typically targeting the spleen and higher values favoring lung or liver delivery. However, pancreas‐targeting LNPs had not been previously characterized. Using TNS assay, we found that the apparent pKa of the pancreas‐targeting C‐CholF3 LNPs is 7.31, compared to a pKa of 6.71 for the liver‐targeting C12‐200 LNPs (Figure S15, Supporting Information). To expand the therapeutic applicability of C‐CholF3 LNPs beyond mRNA delivery, we evaluated their ability to deliver other clinically relevant nucleic acid cargoes, such as plasmid DNA (pDNA) and circular mRNA (circ mRNA), which are increasingly being explored for long‐term expression and enhanced molecular stability, respectively.^[^ ^49^, ^50^, ^51^, ^52^ ^]^ Following intravenous administration of C‐CholF3 LNPs containing either circ mRNA or pDNA, we observed the average total flux of 8.41 × 10⁷ for circ mRNA and 1 × 10⁸ for pDNA with over 99% selectivity for both (Figure S16, Supporting Information), highlighting broad compatibility of the formulation to different nucleic acids. The ability to deliver multiple forms of genetic material is essential for expanding the therapeutic potential of pancreatic gene delivery. Plasmid DNA allows long‐term expression of therapeutic genes, and circular mRNA offers enhanced stability with reduced immunogenicity compared with linear mRNA. Overall, our findings indicate that C‐CholF3 LNPs are a safe and highly effective method for delivering pancreas‐targeted nucleic acid therapeutics with significant implications for pancreatic cancer and diabetes therapies.

**Figure 6:**
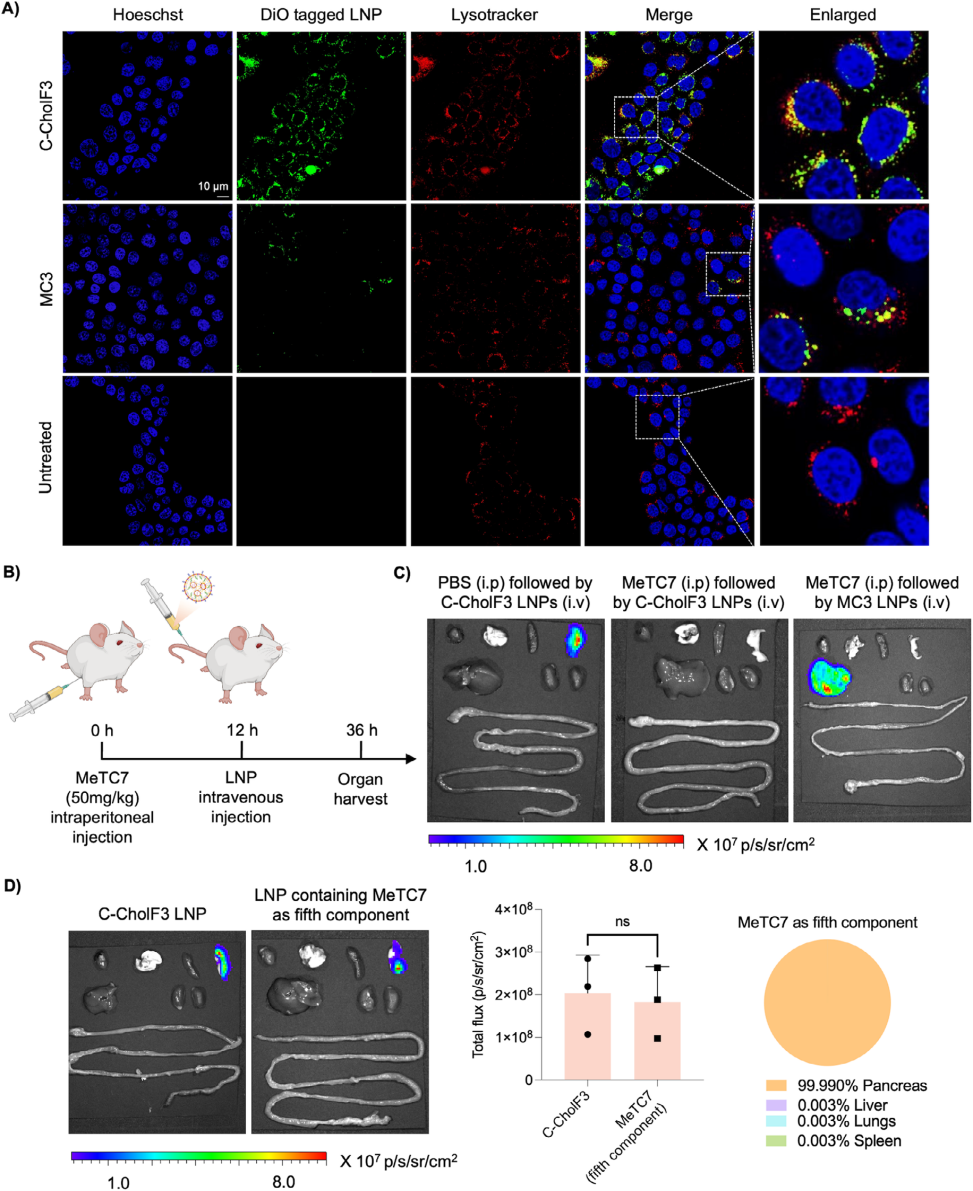
Mechanistic insights into endogenous targeting of C‐CholF3 ENDO LNPs to the pancreas. A) Confocal microscopy images showing the cellular uptake and intracellular trafficking of DiO‐labeled C‐CholF3 and MC3 LNPs in BxPC‐3 cells. Cells were stained with LysoTracker Red (endosomes/lysosomes) and Hoechst 33342 (nuclei) and imaged 2 h post‐treatment to visualize nanoparticle localization. Scale bars are 10 µm. Images were captured at 60× magnification. B) Schematic representation of VDR blockade using MeTC7. Mice were administered 50 mg kg^−1^ of MeTC7 intraperitoneally, and 12 h later, mRNA LNPs were administered intravenously. C) To investigate the role of VDR, mice were pre‐administering with VDR antagonist MeTC7 or PBS (control) 12 h before LNP administration. Representative IVIS images at 24 h post‐injection of LNPs (0.5 mg kg^−1^) in C57BL/6 mice (*n* = 4 biologically independent mice). Organs are arranged left to right as: heart, lung, spleen, pancreas, liver, kidneys and intestines. D) Representative IVIS images, graphical representation of total flux and pie charts illustrating the percentage of protein expression occurring per organ 24 h post‐injection of C‐CholF3 LNPs formulated with MeTC7 as the fifth component in place of cholecalciferol. Mice were treated intravenously at a dose of 0.5 mg kg^−1^ (*n* = 3 biologically independent mice, +‐ SD, *p < 0.05, **p < 0.01, ***p < 0.001. NS, not significant, two‐tailed unpaired t‐test). Organs are arranged left to right as: heart, lung, spleen, pancreas, liver, kidneys and intestines.

We next aim to elucidate the mechanism by which C‐CholF3 ENDO LNPs enable pancreas‐selective delivery. To achieve this, we first investigated their cellular uptake and trafficking in BxPC‐3 human pancreatic cancer cells. Using confocal microscopy, we monitored the internalization and intracellular localization of DiO‐labeled C‐CholF3 and MC3 LNPs. At 2 h post‐treatment, C‐CholF3 LNPs exhibited pronounced colocalization with LysoTracker‐labeled endo/lysosomal vesicles, indicating effective internalization and endosomal trafficking (**Figure** **6**A; Figures S17–S19, Supporting Information). Notably, BxPC‐3 cells treated with C‐CholF3 displayed significantly higher DiO fluorescence compared to MC3‐treated cells, suggesting enhanced cellular uptake potentially due to receptor‐mediated internalization pathways. Many LNPs have been shown to achieve liver and spleen tropism by forming an ApoE‐rich protein corona and a β2‐glycoprotein I (β2‐GPI)‐rich protein corona, respectively.^[^ ^53^, ^54^, ^55^ ^]^ Given that C‐CholF3 contains cholecalciferol as the fifth component in the LNP formulation, we hypothesized that interaction with the VDR may lead to selective uptake by pancreatic cells.

We then investigated the proposed mechanism underlying pancreas tropism in vivo. We employed MeTC7 for this purpose, which is a potent and selective antagonist of VDR.^[^ ^56^, ^57^, ^58^ ^]^ By pre‐administering MeTC7 before LNP treatment, we aimed to block the VDR from facilitating tissue‐selective delivery. This pharmacological strategy enables an acute disruption of VDR‐mediated processes without requiring genetic knockout models, offering valuable mechanistic insight into the role of VDR‐dependent pancreatic delivery of C‐CholF3 LNPs. Twelve hours prior to intravenous injection of C‐CholF3 ENDO LNPs encapsulating Fluc mRNA (0.5 mg kg^−1^), we pretreated C57BL/6 mice intraperitoneally with MeTC7 (50 mg kg^−1^) or PBS as control (Figure 6B). IVIS imaging 24 h post‐injection revealed robust luciferase expression in the pancreas of PBS‐treated mice, confirming selective tropism of C‐CholF3 (Figure 6C; Figure S20, Supporting Information). In contrast, mice pretreated with MeTC7 displayed a complete loss of pancreatic signal, supporting the requirement of functional VDR for tissue‐selective delivery and indicating that MeTC7 disrupts the interaction between VDR and C‐CholF3 LNPs. Moreover, MC3 LNPs delivered to MeTC7‐treated mice continued to exhibit strong hepatic protein expression, consistent with the known mechanism of ApoE‐mediated liver targeting. Similarly, C12‐200 LNPs also exhibited liver accumulation following intravenous injection in MeTC7‐treated mice, indicating that MeTC7 treatment does not interfere with ApoE‐mediated hepatic uptake (Figure S21, Supporting Information). These findings underscore the mechanistic difference between liver‐targeting LNPs and pancreas‐targeting LNPs, emphasizing the critical role of VDR in C‐CholF3‐mediated delivery. To further investigate the role of VDR and assess whether its potential application in targeting could extend beyond cholecalciferol to other VDR binding molecules, we replaced cholecalciferol with MeTC7 as a fifth component in pancreas targeting formulation and evaluated its effect on pancreas tropism in vivo. MeTC7 LNPs were intravenously administered at a dose of 0.5 mg kg^−1^, and 24 h post‐injection, we observed selective and robust luciferase expression in the pancreas (Figure 6D; Figure S22, Supporting Information). Here, MeTC7 is embedded within the LNP and interacts with VDR in a manner analogous to cholecalciferol, thereby promoting efficient pancreatic targeting. This provides further evidence for a direct interaction between the LNPs and VDR, supporting a novel VDR‐mediated delivery mechanism. By exploiting the tissue‐resident or overexpressed receptors through rational lipid design, such as incorporating endogenous ligand cholecalciferol, it is possible to achieve organ‐selective gene delivery with high specificity. This receptor‐ligand matching approach paves the way for rational designs of LNPs to other extrahepatic tissues by aligning lipid ligands with tissue‐specific receptors.

**Figure 7:**
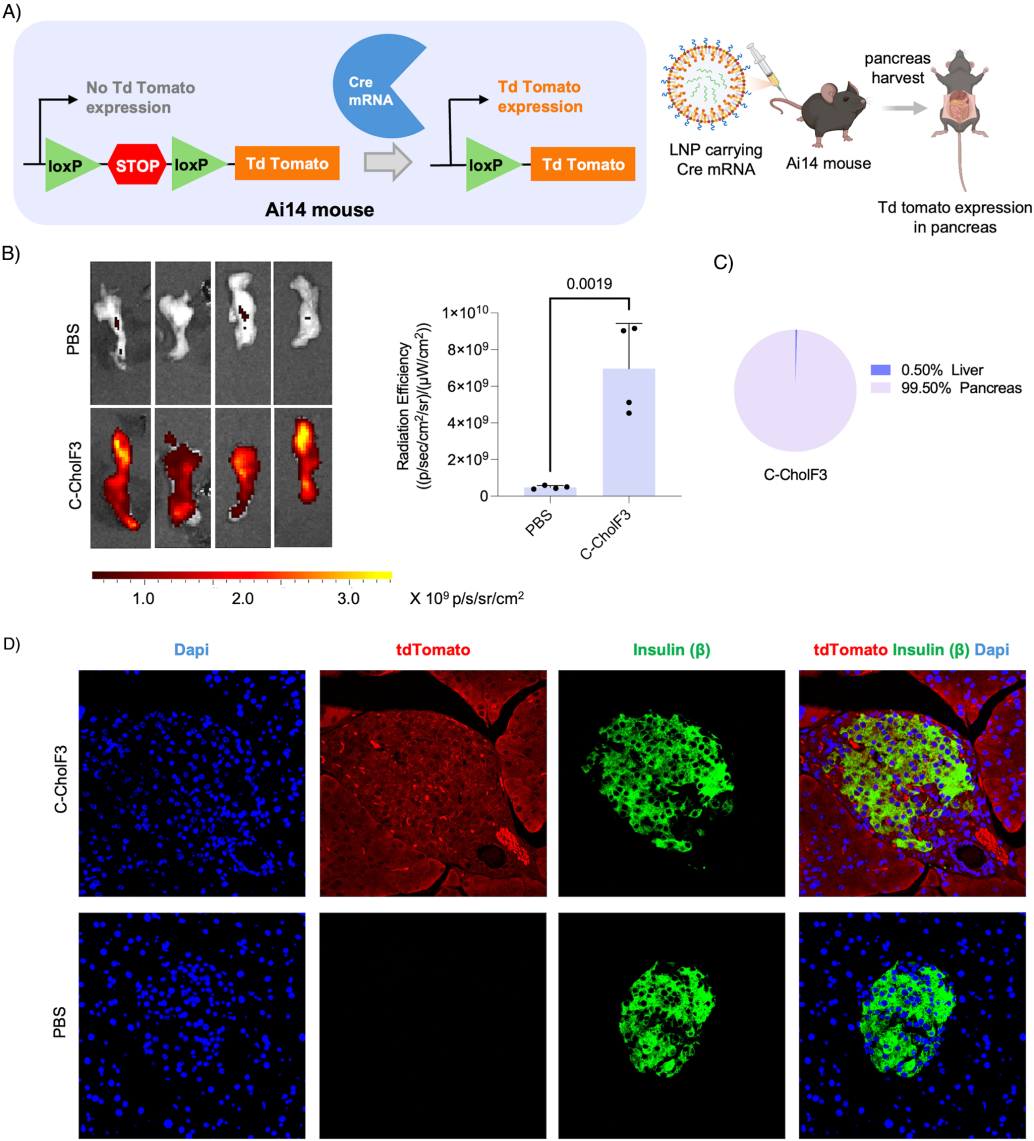
Efficient and tissue‐specific tdTomato expression in the pancreas with C‐CholF3 ENDO LNPs. A) Schematic illustration of Cre mRNA delivery and subsequent Cre‐mediated removal of the stop cassette, leading to the activation of tdTomato expression in the Ai14 Cre‐loxP mouse model. LNPs formulated with Cre mRNA were injected intravenously and the pancreas was imaged using the IVIS. B) Representative tdTomato expression in the pancreas 120 h post‐injection and graphical representation of total flux of C‐CholF3 LNPs containing Cre mRNA, and PBS treated control injected intravenously to Ai14 mice at a dose of 1.5 mg kg^−1^ (*n* = 4 biologically independent mice, ± SD, ^*^ *p* < 0.05, ^**^ *p* < 0.01, ^***^ *p* < 0.001. NS, not significant, two‐tailed unpaired t‐test). C) Pie charts illustrating the percentage of protein expression occurring in pancreas and liver after intravenous injection of Cre mRNA containing C‐CholF3 LNPs. (*n* = 4 biologically independent mice). D) Representative immunofluorescent images of pancreatic sections 120 h post‐injection with C‐CholF3 LNPs encapsulating Cre mRNA and with PBS (control) via intravenous injection in Ai14 mice at a dose of 1.5 mg kg^−1^. Insulin antibody was used to stain the β‐cells, and DAPI was used to label the nuclei. Images were captured at 40× magnification from three biologically independent mice.

Having demonstrated that C‐CholF3 can deliver mRNA to the pancreas, we next utilized the Ai14 mouse model to assess the tissue‐specific gene‐editing capability of C‐CholF3 LNPs. The Ai14 mice are genetically engineered and contain a construct designed for Cre‐mediated gene editing, featuring a LoxP‐stop‐LoxP cassette downstream of the CAG promoter. After successful delivery of Cre recombinase, the stop cassette is excised, activating tdTomato fluorescence in the targeted cells.^[^ ^59^, ^60^ ^]^ Thus, the system simplifies the identification and quantification of the gene‐edited cells, as the cells with Cre mRNA expression exhibit characteristic red tdTomato fluorescence in a tissue‐specific manner (**Figure** **7**A). We formulated the C‐CholF3 LNPs with Cre recombinase mRNA and intravenously injected them into the Ai14 mice at 1.5 mg kg^−1^. After 120 h, fluorescence imaging revealed robust tdTomato expression in the pancreas (Figure 7B) with more than 99% selectivity (Figure 7C; Figure S23, Supporting Information). Minimal fluorescence was observed in other tissues, such as the liver, where LNP uptake and Cre expression were not statistically significant compared to those in the pancreas (Figure S23, Supporting Information). This suggests that the formulation may be applicable to other animal models. Following IVIS detection of tdTomato, we performed immunofluorescence (IF) analysis to understand which cells were undergoing tissue‐specific gene editing. Staining with insulin antibodies identified tdTomato fluorescence within insulin‐positive cells, indicating effective gene editing in pancreatic β cells by C‐CholF3 ENDO LNPs (Figure 7D; Figures S24 and S25, Supporting Information). However, this delivery is not exclusive to β‐cells. These results show that this delivery system can modulate gene expression in major pancreatic cell types, which makes it promising for targeted treatments in pancreatic diseases.

To better evaluate the clinical translatability of C‐CholF3 LNPs, we compared their safety profile with clinically approved MC3 formulation (Onpattro). We assessed in vivo transfection efficiency and toxicity profiles of C‐CholF3 and MC3 at 4 and 24 h post‐injection. IVIS imaging at 4 h (Figure S26A, Supporting Information) demonstrated early pancreatic accumulation of C‐CholF3, with an average total flux of 3.52 × 10^6^, and no redistribution following liver clearance. In contrast, MC3 predominantly accumulated in the liver, exhibiting a higher average total flux of 2.26 × 10^7^. At 24 h, IVIS images (Figure S26B, Supporting Information) confirmed enhanced pancreatic transfection with C‐CholF3 (average total flux of 3.47 × 10^7^) compared to MC3 (average total flux of 3.24 × 10^7^). Safety evaluations indicated that C‐CholF3 exhibited lower serum levels of liver enzymes ALT and AST (Figure S27A, Supporting Information) compared to MC3, similar kidney function parameters were observed for both C‐CholF3 and MC3 (Figure S27B, Supporting Information), and a more favorable cytokine profile (Figure S27C, Supporting Information). Notably, IL‐6 levels were significantly lower with C‐CholF3, whereas IL‐1β levels were higher than those observed with MC3, overall supporting a superior safety profile for C‐CholF3.

## 3 Conclusion

In summary, we report that the addition of endogenous cholecalciferol as a fifth component successfully redirects traditional four‐component C12‐200 LNPs from the liver to the pancreas following intravenous administration. Using a comprehensive screening approach, we identified C‐CholF3 LNPs containing cholecalciferol as a fifth component that exhibits robust protein expression in the pancreas with over 99% selectivity and reduced toxicity. We propose that the mechanism underlying pancreas tropism in vivo is driven by an endogenous targeting mechanism involving VDR. Additionally, C‐CholF3 facilitates selective pancreatic delivery of both plasmid DNA and circular mRNA, showcasing its versatility and therapeutic promise. Furthermore, C‐CholF3 showed robust pancreas‐specific tdTomato expression, especially in pancreatic β cells of the Ai14 transgenic mouse model, highlighting the effectiveness of C‐CholF3 LNPs in gene editing. These findings indicate that formulations with the appropriate endogenous fifth component could be utilized to redirect therapies to extrahepatic space with minimal toxicity, potentially advancing the development of gene therapy or personalized medicine for pancreatic diseases with the capability of repeat administration.

## 4 Experimental Section

### Animal Experiments

All animal procedures were conducted in accordance with guidelines and approval from the Institutional Animal Care and Use Committee (IACUC) at the University of Las Vegas, Nevada (protocol #01218). Female and male C57BL/6J mice (6–8 weeks old, ≈20 g) and B6.Cg‐Gt(ROSA)26Sortm14(CAG‐tdTomato)Hze/J mice (6–8 weeks old, ≈20 g) were obtained from Jackson Laboratory (Bar Harbor, ME, USA). Mice were injected with LNPs formulated with FLuc mRNA at a dose of 0.5 mg kg^−1^ via the lateral tail vein. For mechanism studies, female C57BL/6J mice (6–8 weeks old, ≈20 g) were intraperitoneally injected with 50 mg kg^−1^ of MeTC7 (MedChemExpress, USA). After 12 h of administration of MeTC7, mice were intravenously injected via the lateral tail vein with FLuc mRNA LNPs at a dose of 0.5 mg kg^−1^. A D‐luciferin solution (30 mg mL^−1^ in 1X PBS; PerkinElmer) was prepared, and mice were injected intraperitoneally with 130 µL of this solution after 24 h (for intravenous injections). After a 10‐min incubation, the mice were euthanized using CO_2_, and organs (liver, spleen, kidney, pancreas, heart, lung) were harvested and imaged with an in vivo imaging system (IVIS; PerkinElmer, Waltham, MA, USA). Luminescence flux was quantified using Living Image Software (PerkinElmer). For toxicity assessment, blood samples were collected via cardiac puncture 24 h after treatment. Mice were then sacrificed by CO_2_ asphyxiation, and organs were collected. Blood samples were allowed to coagulate for 20 min at room temperature and centrifuged at 2000 × g for 20 min at 4 °C to obtain high‐quality of serum. Serum liver enzyme levels were measured by VRL Animal Health Diagnostics and the cytokine panel was performed by Eve Technologies Corp. (Calgary, Alberta). For Ai14 mice experiments, fluorescence was quantified at an excitation/emission of 554/581 nm. Regions of interest (ROIs) of a constant size were placed around each organ's image, and total luminescence flux and radiant efficiency were reported as mean ± standard deviation.

### Statistics

Statistical analysis of the results was performed using One‐Way ANOVA followed by Bonferroni post‐hoc analysis or two‐tailed unpaired t‐test to compare multiple replicates means using Prism 10 (GraphPad). Differences were considered significant when *p* < 0.05.

## Conflict of Interest

I.I. and C.B. have filed a patent application based on this work. The other authors declare no conflict of interest.

## Author Contributions

I.I. and C.B. conceived the project, designed the experiments, and wrote the manuscript. I.I. synthesized the LNPs, performed all the in vitro and in vivo experiments, and analyzed data. L.P. assisted I.I. with the high‐throughput screen and N.T. with immunofluorescence optimization. A.S. helped with confocal imaging. D. Y. obtained cryogenic transmission electron micrographs of LNPs. P.G. and S.P. provided valuable comments. C.B. directed the research.

## Supporting information



Supporting Information

## Data Availability

The data that support the findings of this study are available from the corresponding author upon reasonable request.
