# Supplementary material for: Reengineering Endogenous Targeting Lipid Nanoparticles (ENDO) for Systemic Delivery of mRNA to Pancreas
Source: Adv Mater. 2025 Jun 12;37(40):2507657. doi: 10.1002/adma.202507657 (PMC12510295; doi:10.1002/adma.202507657)
Supplement: Supplementary file 1 — Supporting Information [file ADMA-37-2507657-s001.pdf]

ADVANCED MATERIALS

Supporting Information

for *Adv. Mater.*, DOI 10.1002/adma.202507657

Reengineering Endogenous Targeting Lipid Nanoparticles (ENDO) for Systemic Delivery of mRNA to Pancreas

*Ivan Isaac, Luv Patel, Nguyen Tran, Amarnath Singam, DongSoo Yun, Prasun Guha, Seungman Park and Chandrabali Bhattacharya**

Supporting Information

Reengineering Endogenous Targeting Lipid Nanoparticles (ENDO) for Systemic Delivery of mRNA to Pancreas

*Ivan Isaac[1], Luv Patel[1], Nguyen Tran[2], Amarnath Singam[4], DongSoo Yun[5], Prasun Guha[2,3], Seungman Park[4,6], Chandrabali Bhattacharya[1,6]**

[1]Department of Chemistry and Biochemistry, University of Nevada Las Vegas, Las Vegas, NV 89154, USA

[2]Nevada Institute of Personalized Medicine, University of Nevada, Las Vegas, Las Vegas, NV 89154, USA

[3]School of Life Sciences, College of Sciences, University of Nevada, Las Vegas, Las Vegas, NV 89154, USA

[4]Department of Mechanical Engineering, University of Nevada, Las Vegas, Las Vegas, NV 89154, USA

[5]Koch Institute for Integrative Cancer Research, Massachusetts Institute of Technology, Cambridge, MA 02139, USA

[6]Interdisciplinary Biomedical Engineering Program, University of Nevada, Las Vegas, Las Vegas, NV 89154, USA

*Correspondence:

Chandrabali Bhattacharya, Department of Chemistry and Biochemistry, University of Nevada Las Vegas, Las Vegas, Nevada 89154, United States, Email: chandra.bhattacharya@unlv.edu

	Ionizable lipid	DOPE	Cholesterol	PEG	5th Component
**F1**	30	16	46.5	2.5	5
**F2**	25	16	46.5	2.5	10
**F3**	20	16	46.5	2.5	15
**F4**	35	16	41.5	2.5	5

Table S1. Summary of LNP formulation ratios used to formulate ENDO LNP library.

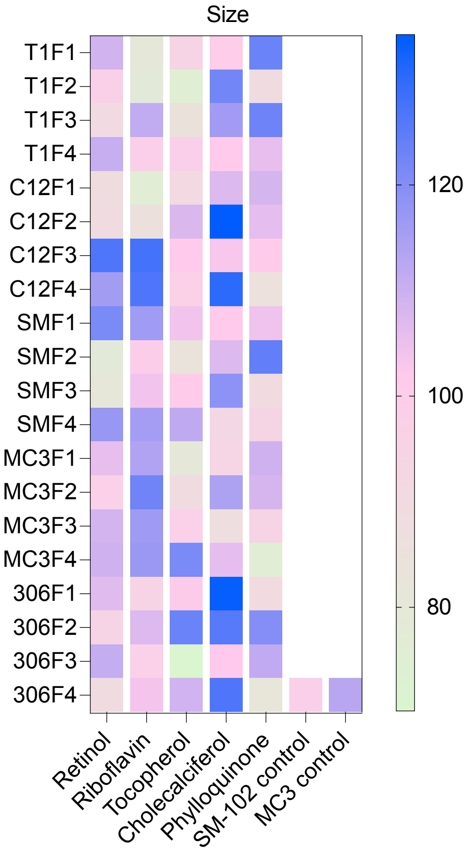

Figure S1. Heatmap representing size (in nm) of ENDO LNPs measured using DLS (n=3). Abbreviation used: T1, THP1; C12, C12-200; SM, SM-102; 306, 306Oi10; F1, Formulation 1; F2, Formulation 2; F3, Formulation 3; F4, Formulation 4.

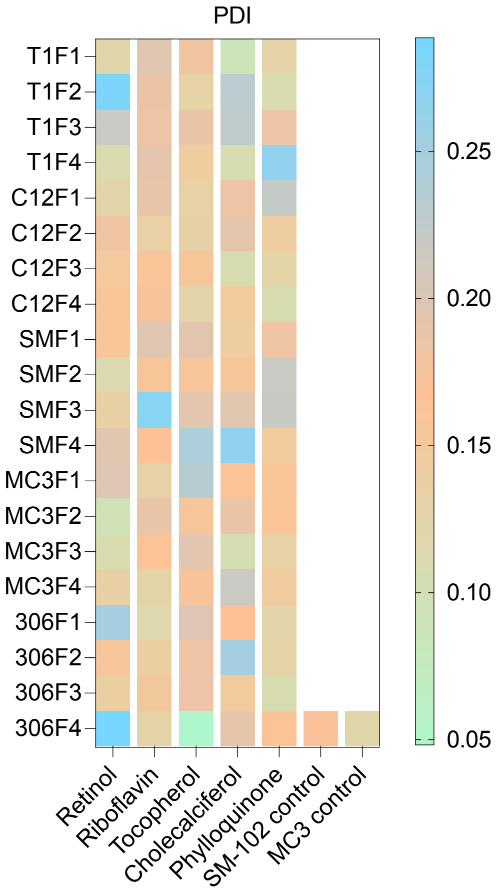

Figure S2. Heatmap representing PDI of ENDO LNPs measured using DLS (n=3). Abbreviation used: T1, THP1; C12, C12-200; SM, SM-102; 306, 306Oi10; F1, Formulation 1; F2, Formulation 2; F3, Formulation 3; F4, Formulation 4.

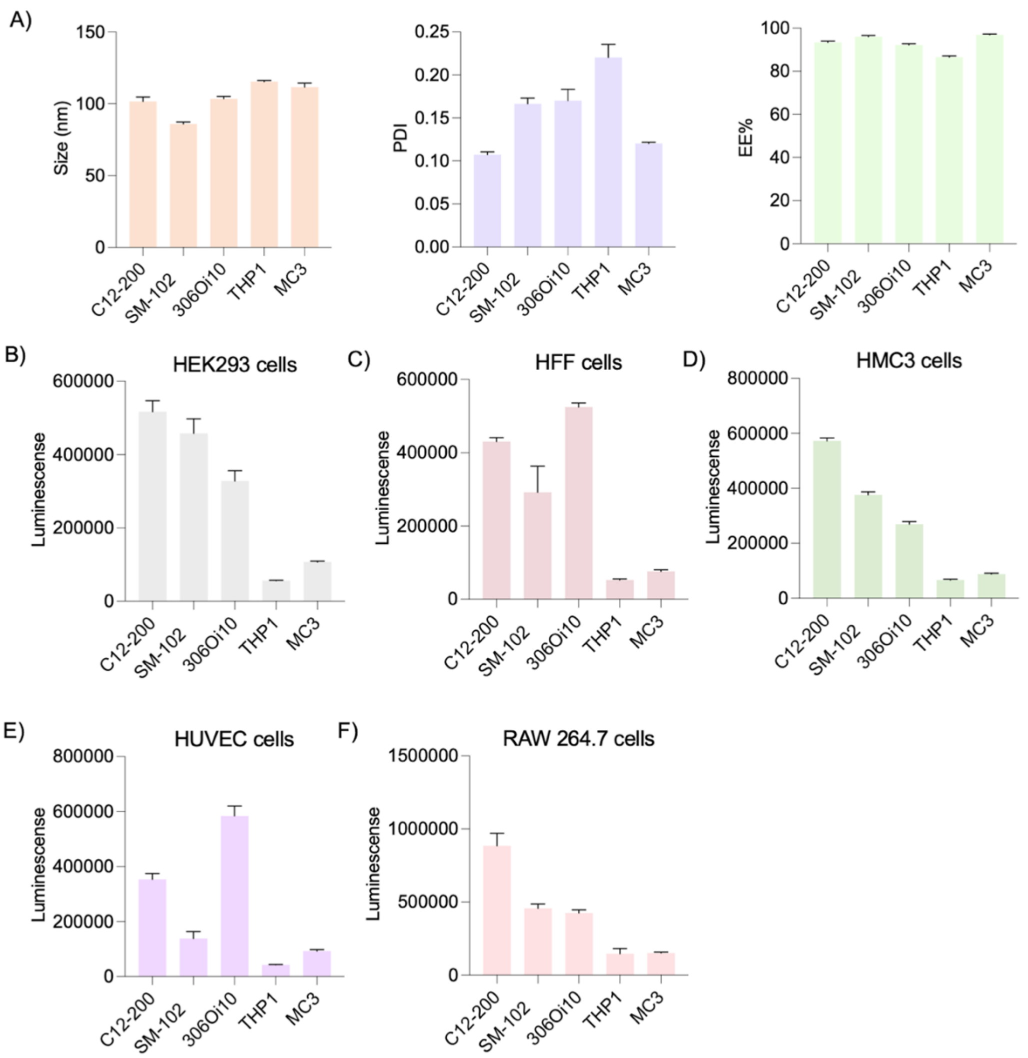

Figure S3. Characterization and transfection efficiency of traditional four component control LNPs. A) The size (nm), polydispersity index (PDI), and encapsulation efficiency (EE%) of traditional four component control LNPs. B) *In Vitro* delivery efficacy of FLuc mRNA traditional four component LNPs in HEK293 cells (125 ng mRNA per well, 96 well plate, n=3). C) *In Vitro* delivery efficacy of FLuc mRNA traditional four component LNPs in HFF cells (125 ng mRNA per well, 96 well plate, n=3). D) *In Vitro* delivery efficacy of FLuc mRNA traditional four component LNPs in HMC3 cells (125 ng mRNA per well, 96 well plate, n=3). E) *In Vitro* delivery efficacy of FLuc mRNA traditional four component LNPs in HUVEC cells (125 ng mRNA per well, 96 well plate, n=3). F) *In Vitro* delivery efficacy of FLuc mRNA traditional four component LNPs in RAW264.7 cells (125 ng mRNA per well, 96 well plate, n=3). Luminescence intensity was quantified 24 h after adding LNPs.

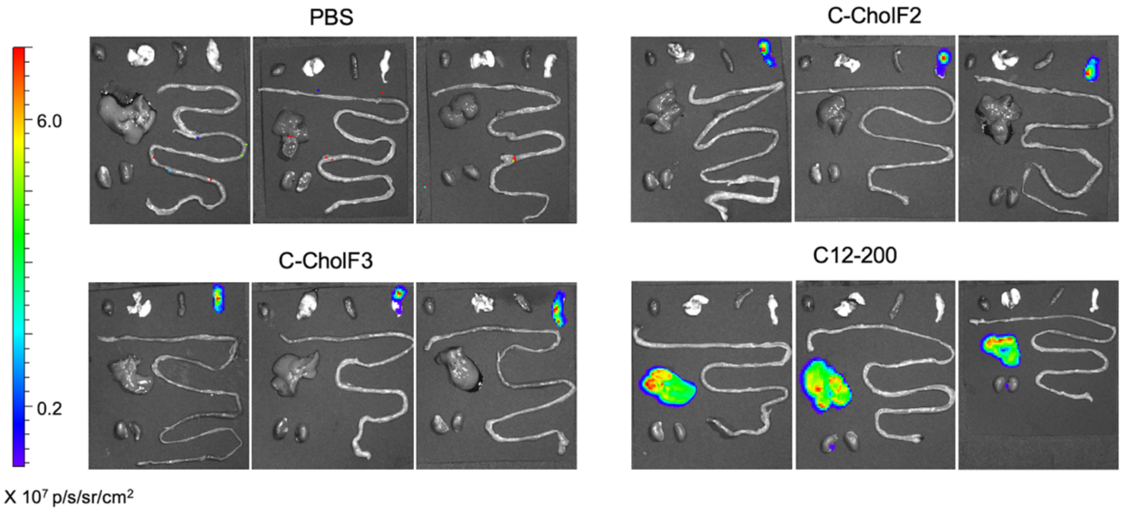

Figure S4. All replicates of IVIS images from **Figure 4B** after administration of C-CholF2 and C-CholF3 LNPs injected intravenously at a dose of 0.5 mg/kg. PBS and C12-200 were also injected (n=3 biologically independent mice). Organs are arranged left to right as: heart, lung, spleen, pancreas, liver, intestines, and kidneys.

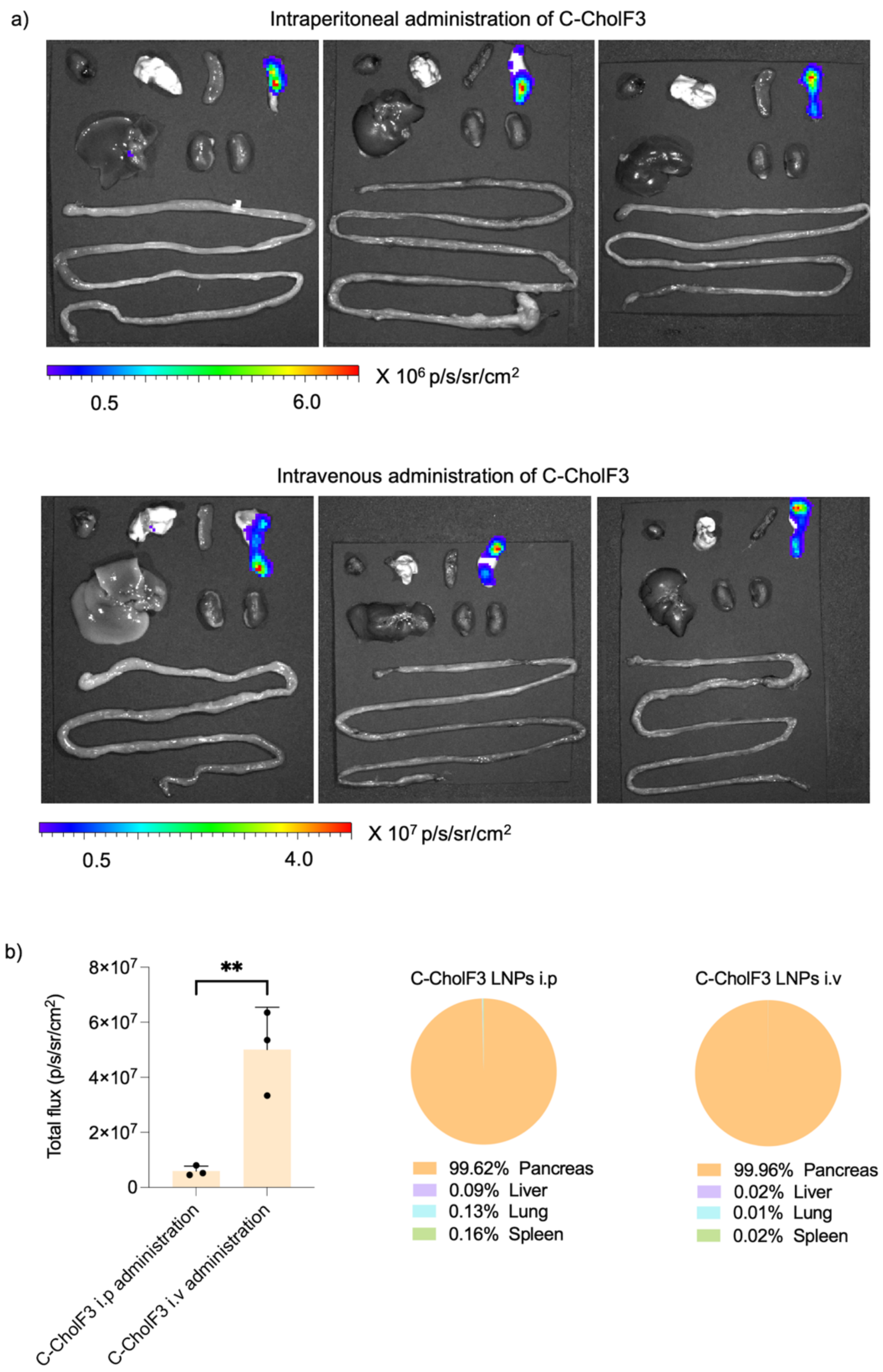

Figure S5. a) Representative IVIS images at 12 h post-injection and graphical representation of total flux of C-CholF3 ENDO LNPs injected intraperitoneally and intravenously at a dose of 0.5 mg/kg. Organs are arranged left to right as: heart, lung, spleen, pancreas, liver, kidneys and intestines. b) Graphical representation of total flux and pie charts illustrating the percentage of protein expression occurring per organ after intraperitoneal and intravenous injection of C-CholF3 ENDO LNPs at a dose of 0.5 mg/kg. (n=3 biologically independent mice, ± SD, *P < 0.05, **P < 0.01, ***P < 0.001, ****P < 0.0001. NS, not significant, one-way ANOVA with Bonferroni post-hoc analysis).

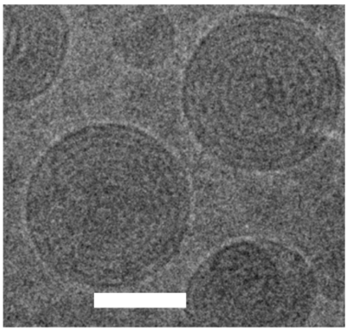

Figure S6. Representative cryo-EM image of C-CholF3 LNPs. Scale bar, 50 nm.

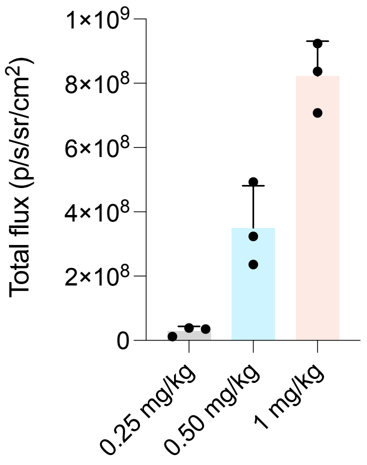

Figure S7. Graphical representation of total flux at 24h post-injection of different doses (0.25, 0.5, and 1 mg/kg) of C-CholF3 LNPs administered intravenously in C57BL/6 mice (n = 3 biologically independent mice).

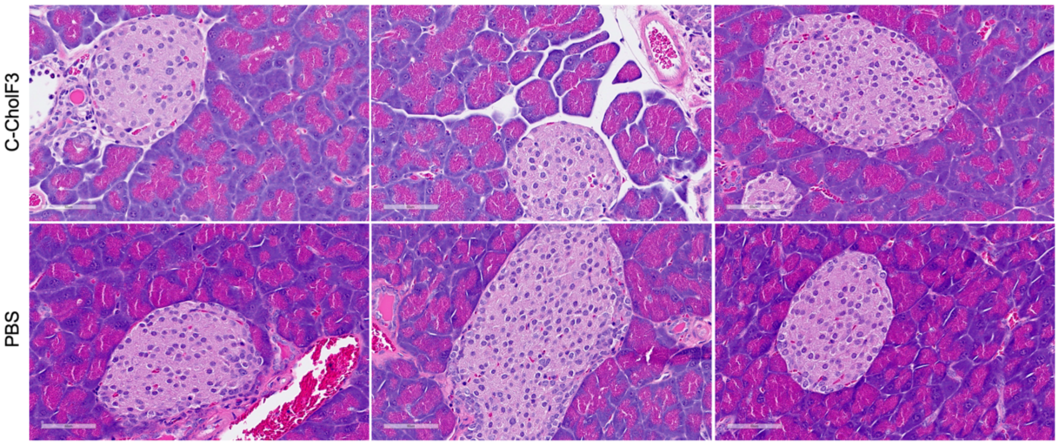

Figure S8. Replicates of H&E staining of the pancreas after 24 h used to generate **Figure 5A.**

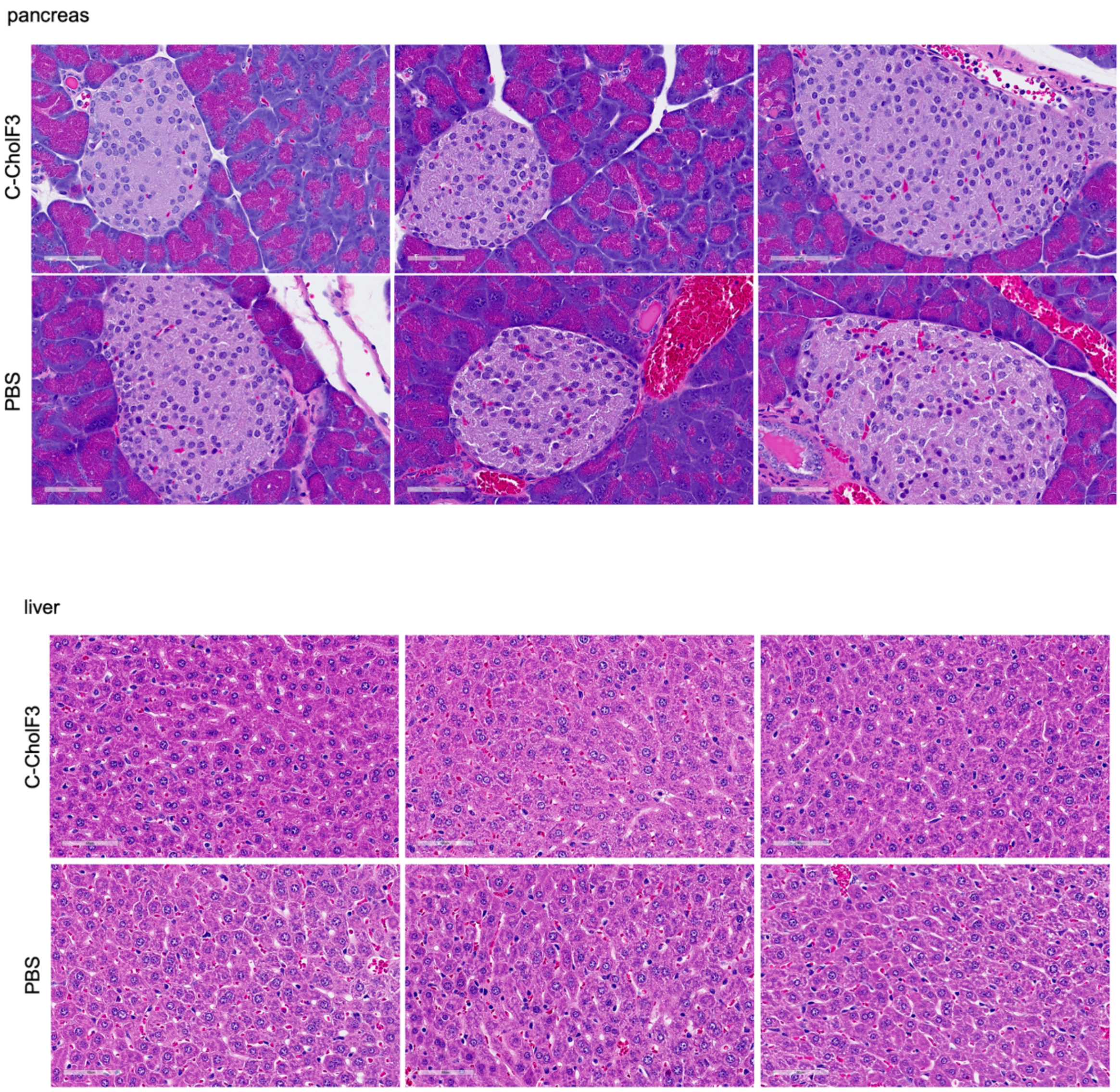

Figure S9. Replicates of H&E staining of the pancreas and liver after 24 h used to generate **Figure 5A.**

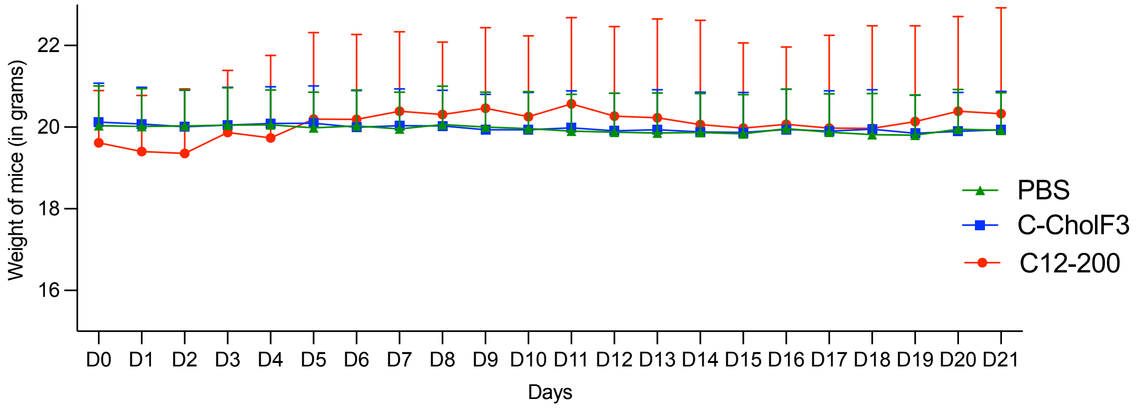

Figure S10. The body weight of mice treated with C-CholF3 and C12-200 LNPs at a dose of 0.5 mg/kg intravenously for 21 days (n=3). PBS was intravenously injected into mice as a control.

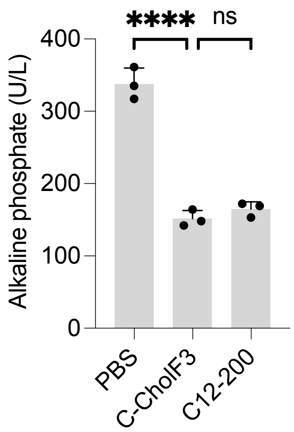

Figure S11. Serum levels of the liver enzyme alkaline phosphatase 24 h after intravenous administration with PBS, C-CholF3, and C12-200 LNPs at a dose of 0.5 mg/kg. (n=3 biologically independent mice, ± SD, *P < 0.05, **P < 0.01, ***P < 0.001, ****P < 0.0001. NS, not significant, one-way ANOVA with Bonferroni post-hoc analysis).

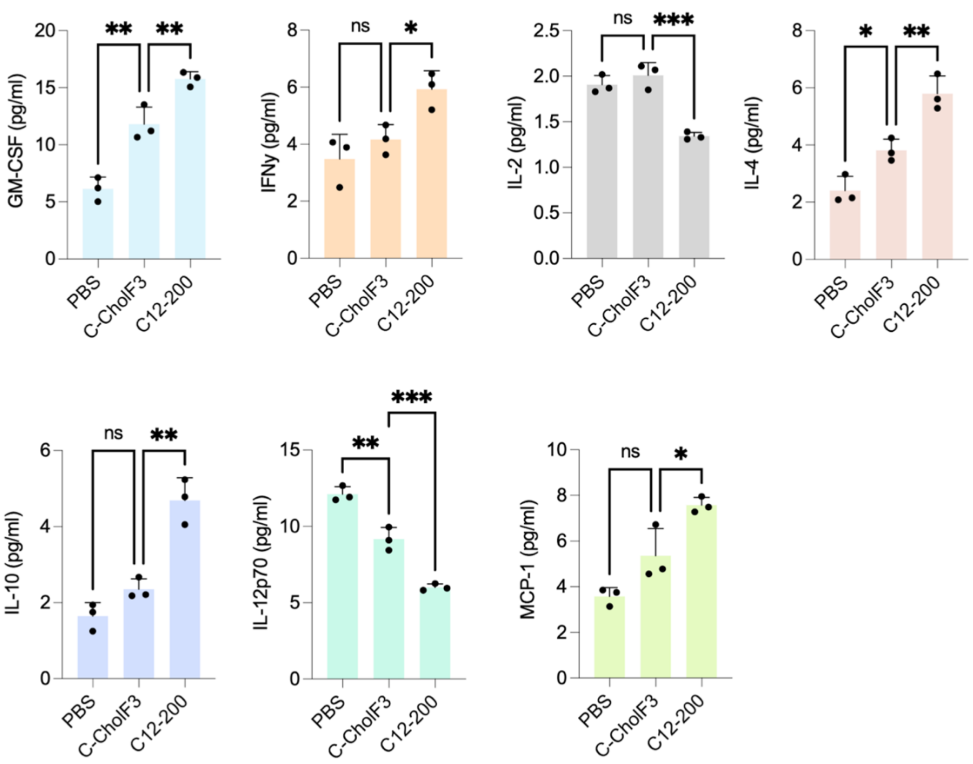

Figure S12. Levels of biomarkers GM-CSF, IFNγ, IL-2, IL-4, IL-10, IL-12P70, and MCP-1 (24 h post-treatment) in mice intravenously treated with C-CholF3 and C12-200 LNPs at a dose of 0.5 mg/kg (n=3 biologically independent mice, ± SD, *P < 0.05, **P < 0.01, ***P < 0.001. NS, not significant, one-way ANOVA with Bonferroni post-hoc analysis). PBS-injected mice were kept as the control group.

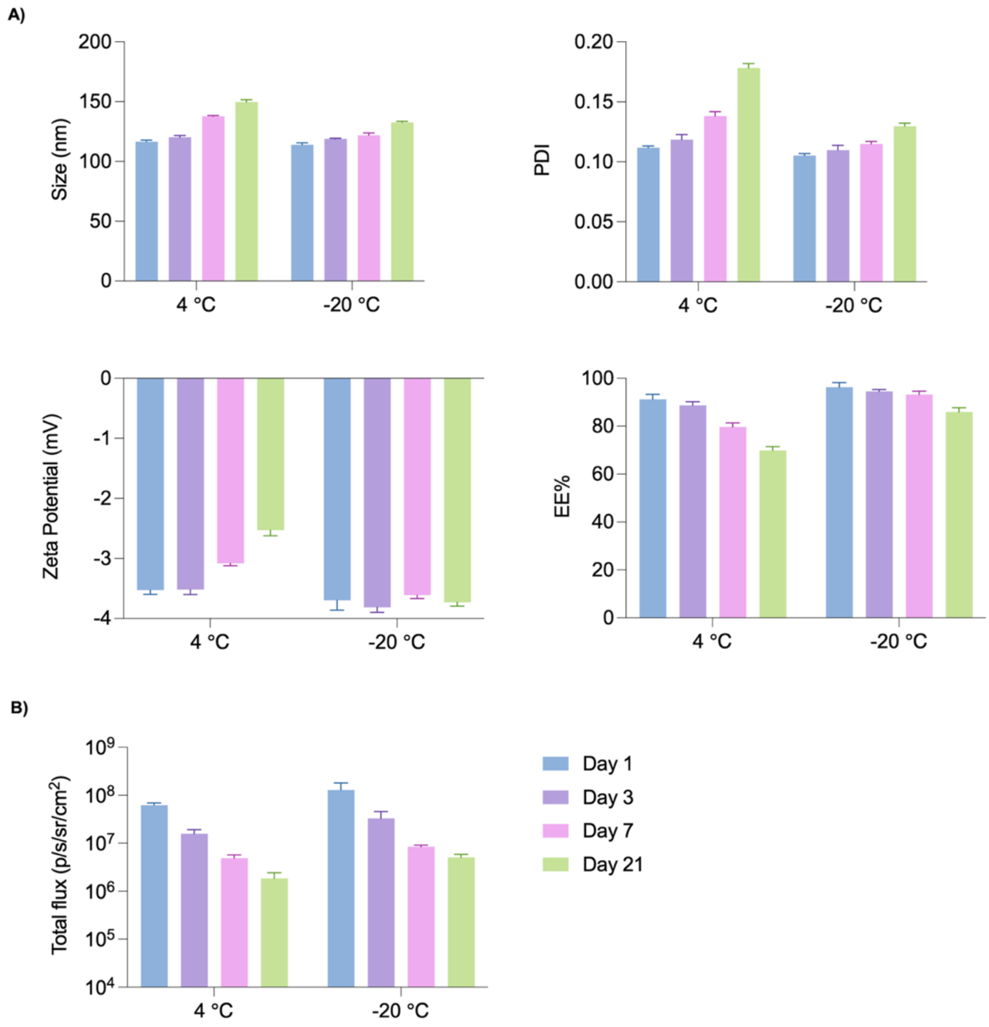

Figure S13. Stability study of C-CholF3 LNPs. A) Size, PDI, and ζ-potential of C-CholF3 LNPs under different storage conditions (4°C and -20°C) for 1, 3, 7, and 21 days. B) Representative IVIS images of total flux 24 h after intravenous injection of FLuc mRNA-loaded C-CholF3 LNPs after being stored at different temperatures for extended time (0.5 mg/kg, n = 3).

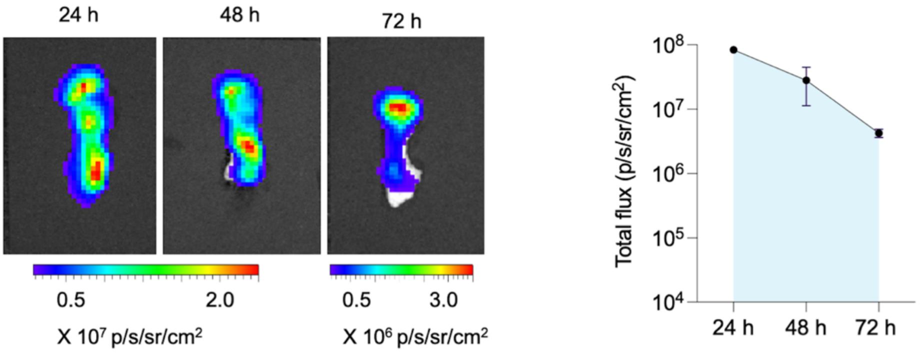

Figure S14. IVIS images and graphical representation of *in vivo* kinetics of FLuc expression following intravenous injection of C-CholF3 LNPs in the pancreas at a dose of 0.5 mg/kg (n = 3 biologically independent mice, ± SD). The luciferase expression was visualized at 24, 48, and 72 hours after injection by IVIS.

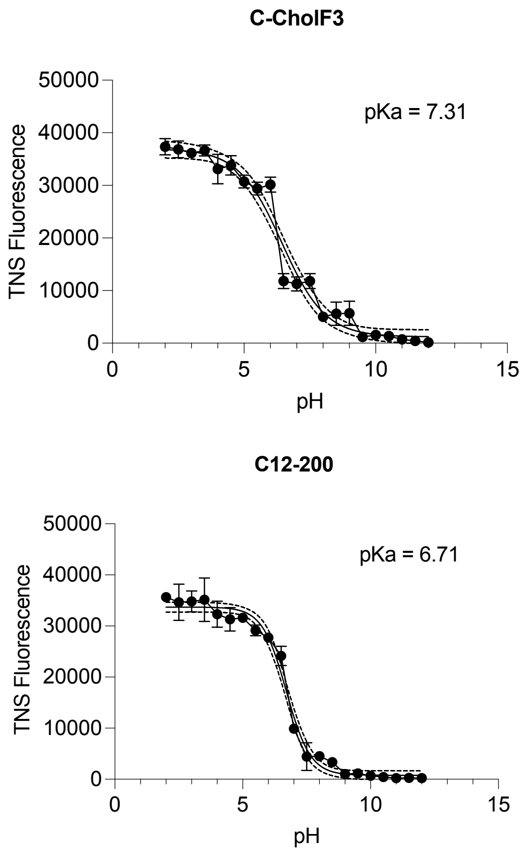

Figure S15. TNS curves for pKa measurements of C-CholF3 and C12-200 LNPs.

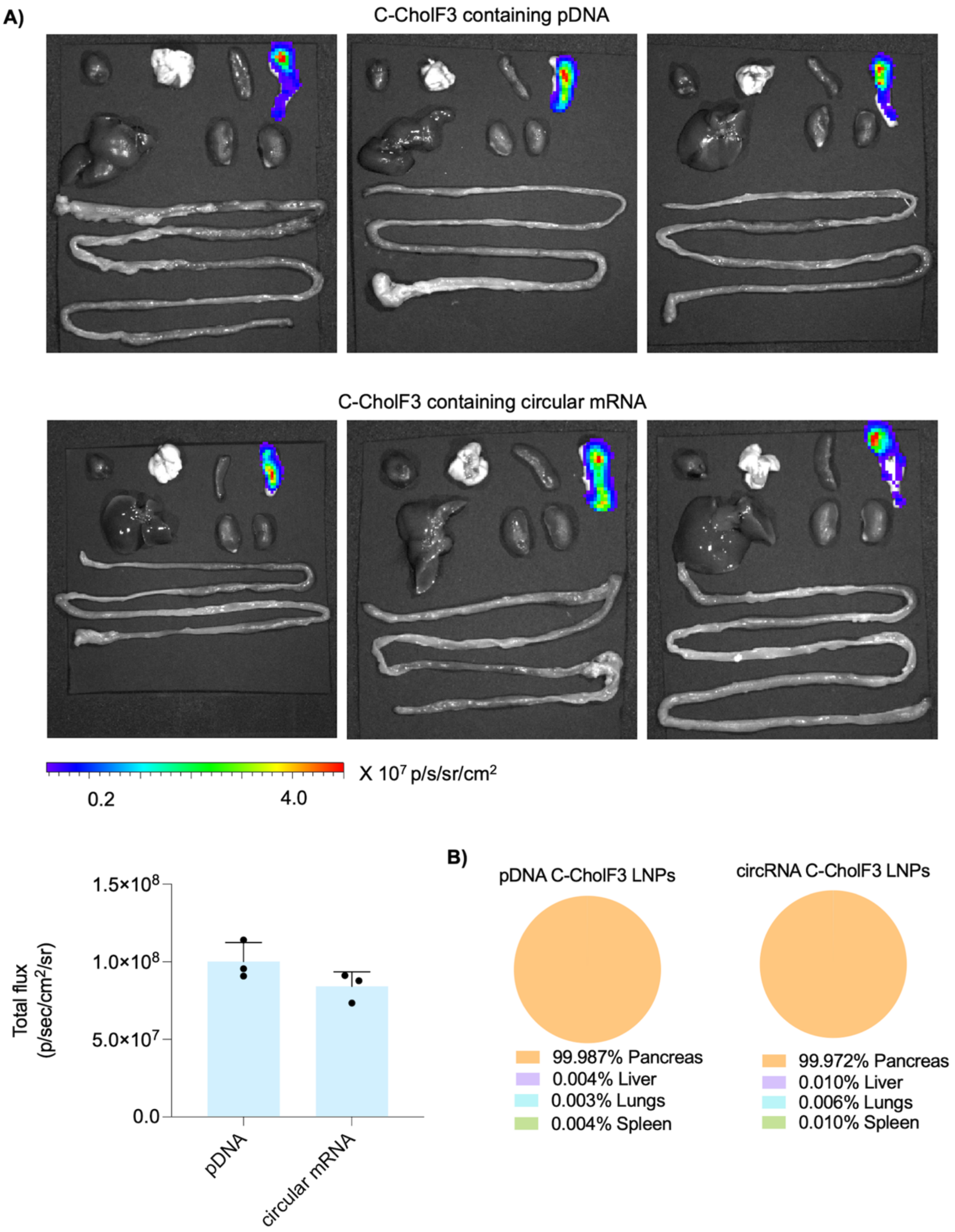

Figure S16. C-CholF3 can deliver plasmid DNA (pDNA) and circular mRNA (mRNA) to the pancreas *in vivo*. A) Representative IVIS images at 24 h post-injection and graphical representation of total flux of C-CholF3 ENDO LNPs formulated with pDNA and circular mRNA injected intravenously at a dose of 0.5 mg/kg (n=3 biologically independent mice). Organs are arranged left to right as: heart, lung, spleen, pancreas, liver, kidneys and intestines. B) Pie charts illustrating the percentage of protein expression occurring per organ after intravenous injection of C-CholF3 ENDO LNPs formulated with pDNA and circular mRNA (n=3 biologically independent mice).

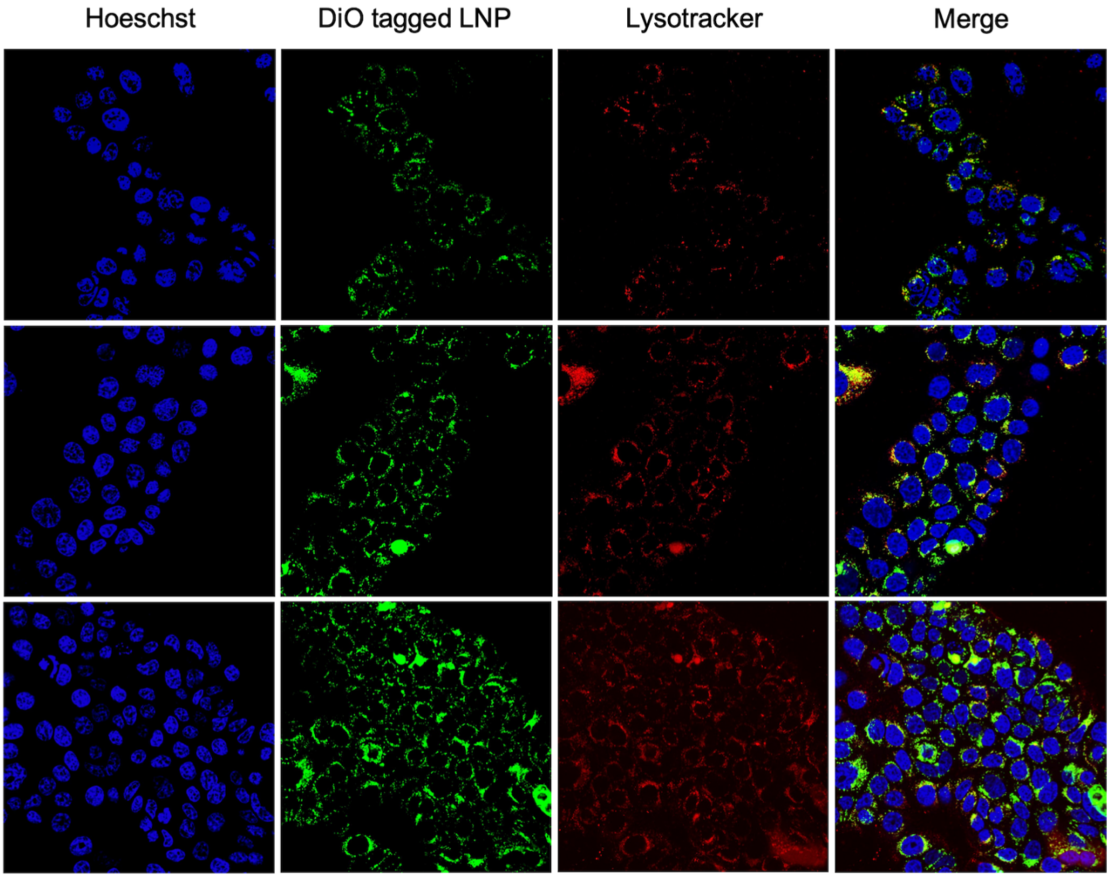

Figure S17. Replicates of confocal microscope images to generate **Figure 6A**. Confocal images of BxPC-3 treated with DiO tagged C-CholF3 ENDO LNPs (green). Cells were stained with LysoTracker Red (endosomes/lysosomes) and Hoechst 33342 (nuclei). Scale bars are 10 µm. Images were captured at 63× magnification.

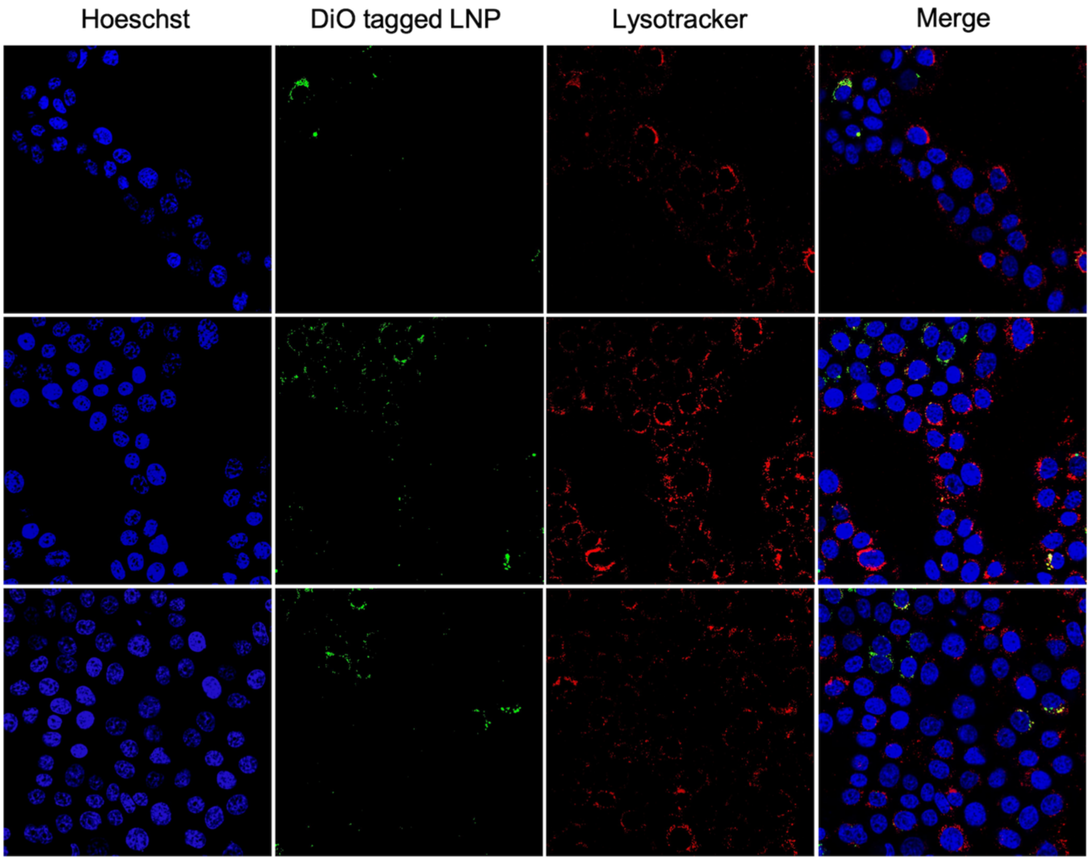

Figure S18. Replicates of confocal microscope images to generate Figure 6A. Confocal images of BxPC-3 treated with DiO tagged MC3 LNPs (green). Cells were stained with LysoTracker Red (endosomes/lysosomes) and Hoechst 33342 (nuclei). Scale bars are 10 μm. Images were captured at 63× magnification.

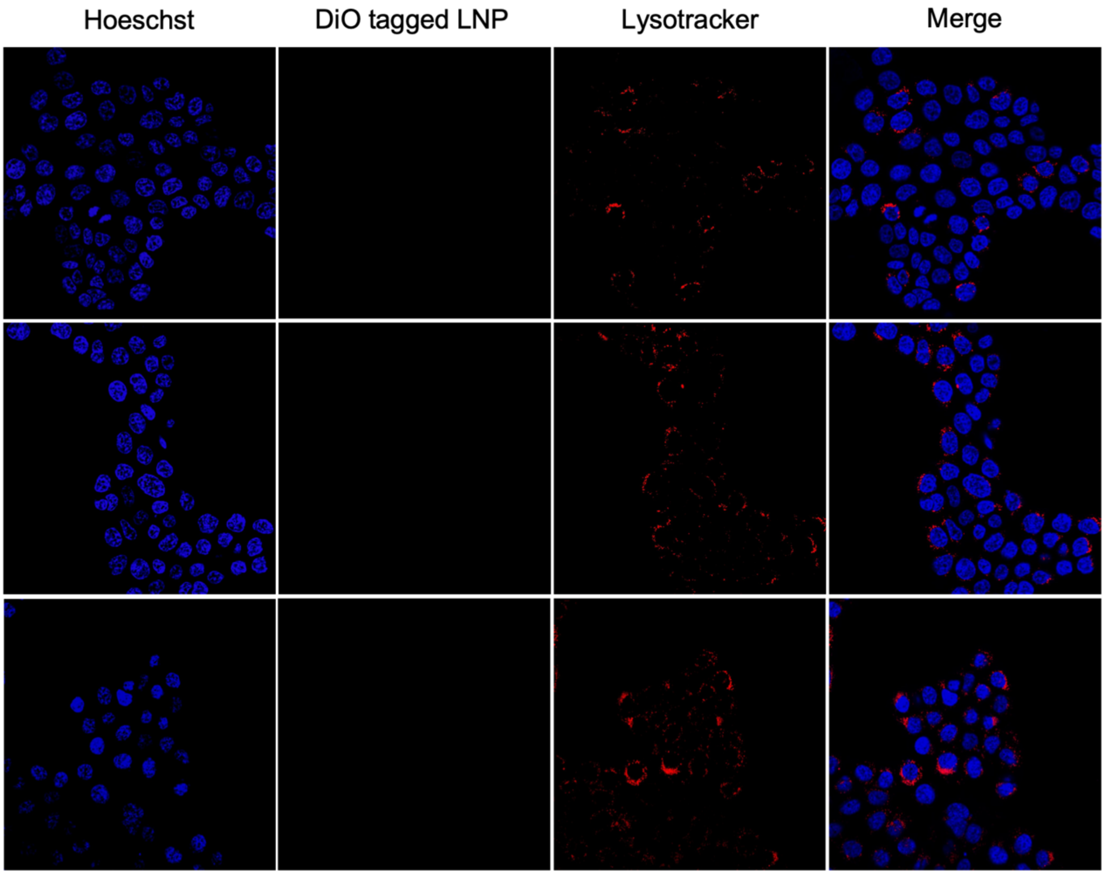

Figure S19. Replicates of confocal microscope images to generate Figure 6A. Confocal images of untreated BxPC-3 cells. Cells were stained with LysoTracker Red (endosomes/lysosomes) and Hoechst 33342 (nuclei). Scale bars are 10 µm. Images were captured at 63× magnification.

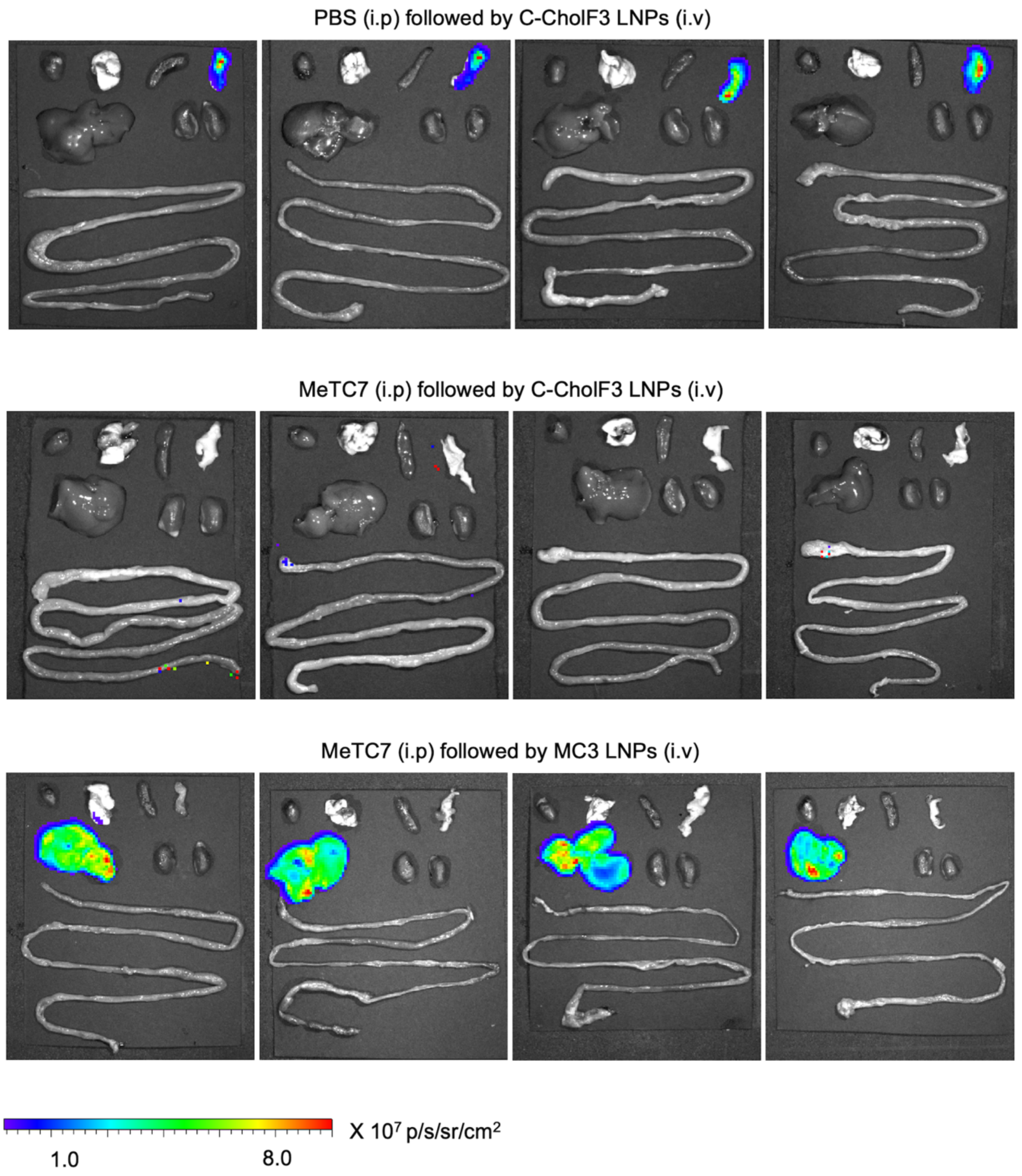

Figure S20. All replicates of IVIS images from **Figure 6D** showing luciferase expression 24 h after intravenous administration of C-CholF3 and MC3 mRNA LNPs (0.5 mg/kg) in C57BL/6 mice. Mice were pretreated intraperitoneally with either 50 mg/kg MeTC7 (VDR antagonist) or PBS (control) 12 h before LNP administration (n = 4 biologically independent mice). Organs are arranged left to right as: heart, lung, spleen, pancreas, liver, kidneys and intestines.

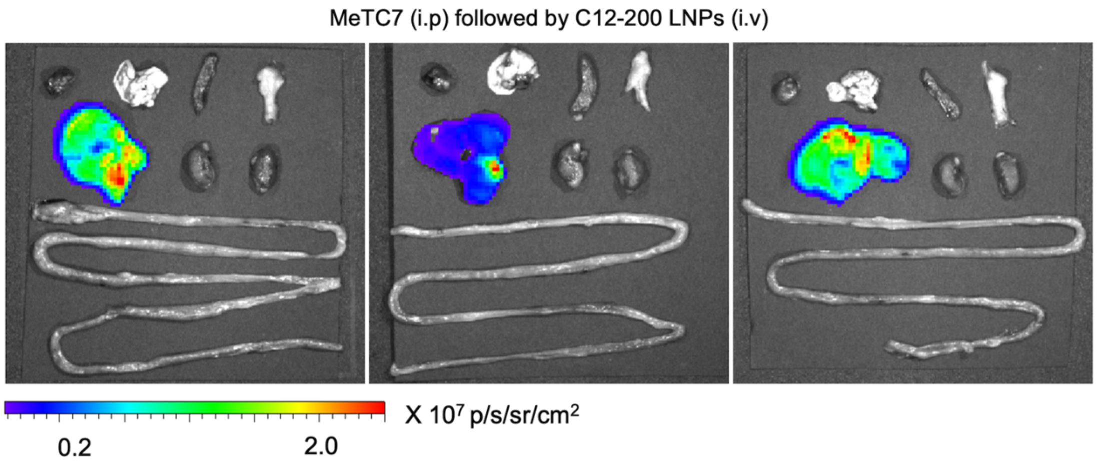

Figure S21. Representative IVIS images at 24 h post-injection of C12-200 LNPs (0.5 mg/kg) in C57BL/6 mice (n = 3 biologically independent mice). Mice were pre-administered with VDR antagonist MeTC7 12 h before LNP administration. Organs are arranged left to right as: heart, lung, spleen, pancreas, liver, kidneys and intestines.

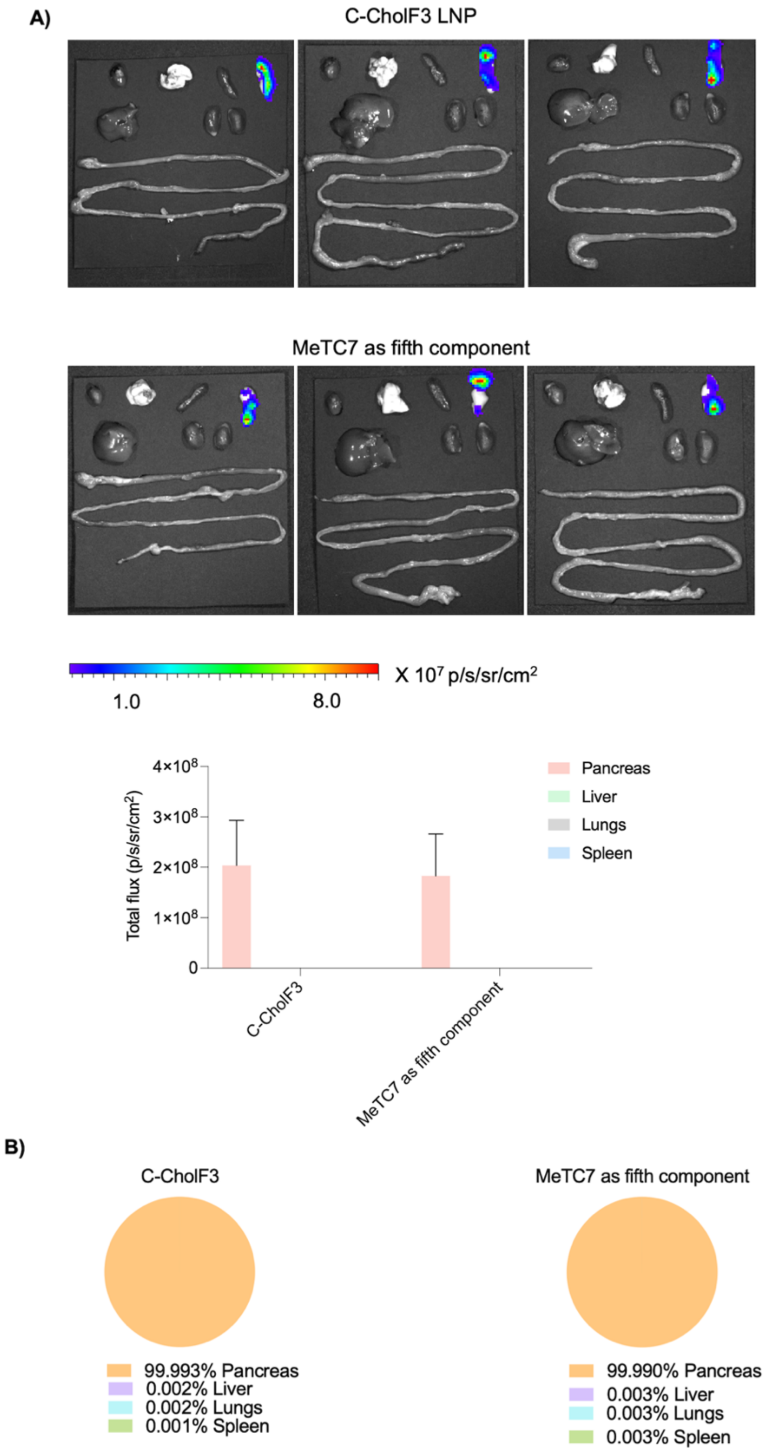

Figure S22. MeTC7 as a fifth component in pancreas targeting formulation can retain pancreas tropism *in vivo*. A) All replicates of IVIS images from **Figure 6E** and graphical representation of total flux of C-CholF3 LNPs and LNPs with MeTC7 as the fifth component 24 h post-injection. Mice were treated intravenously at a dose of 0.5 mg/kg (n = 3 biologically independent mice). Organs are arranged left to right as: heart, lung, spleen, pancreas, liver, kidneys and intestines. B) Pie charts illustrating the percentage of protein expression occurring per organ after intravenous injection of C-CholF3 LNPs and LNPs with MeTC7 as the fifth component (n=3 biologically independent mice).

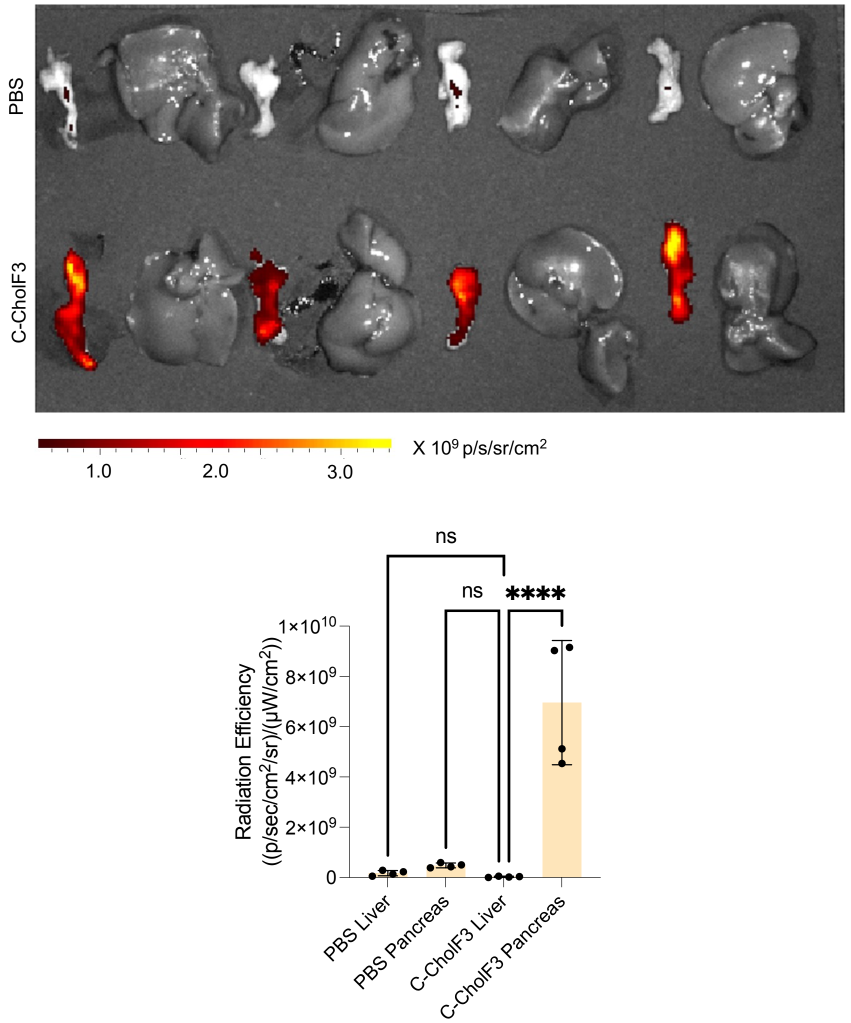

Figure S23. Representative tdTomato expression in pancreas and liver 120h post-injection of C-CholF3 LNPs containing Cre mRNA, and PBS treated control injected intravenously to Ai14 mice at a dose of 1.5 mg/kg (n=4 biologically independent mice, ± SD, *P < 0.05, **P < 0.01, ***P < 0.001. NS, not significant, one-way ANOVA with Bonferroni post-hoc analysis).

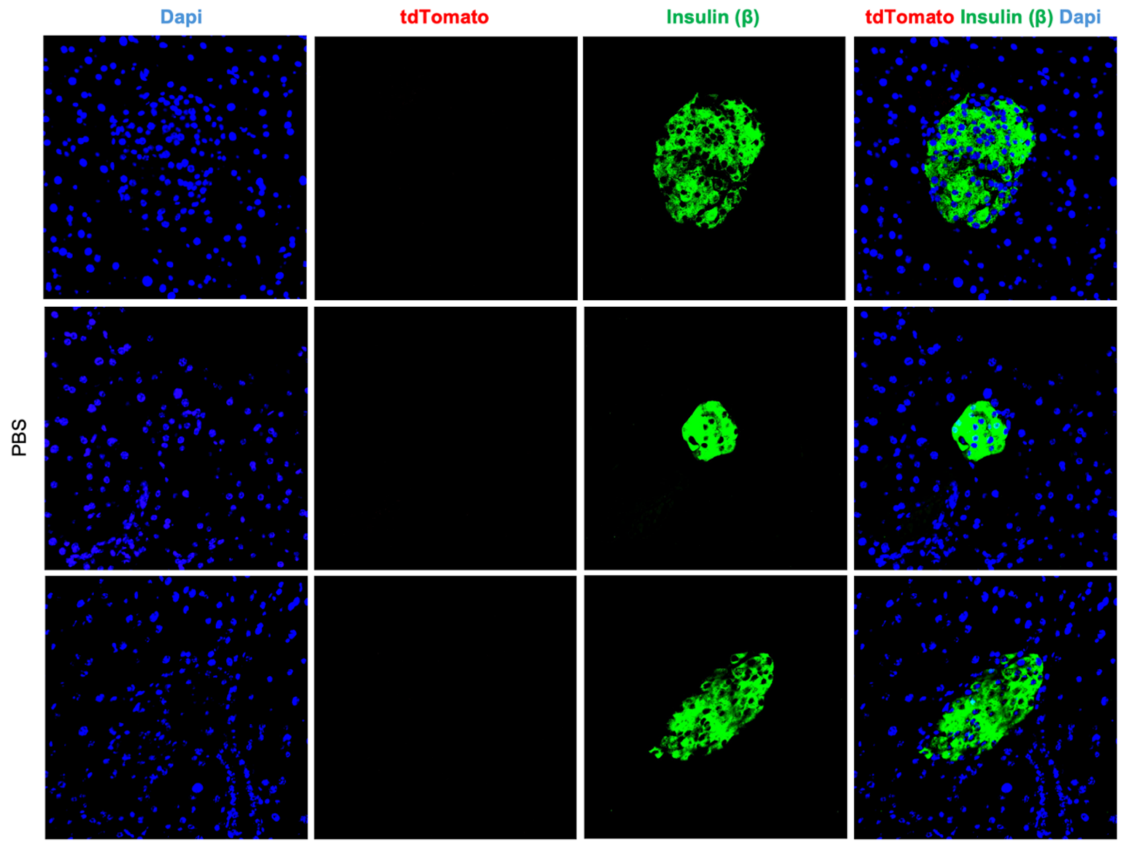

Figure S24. Replicates of images used to generate **Figure 7D**. Immunofluorescent images of pancreatic sections from PBS treatment Ai14 mice. Insulin antibody was used to stain the β-cells, and DAPI was used to label the nuclei. Images were captured at 40× magnification from three biologically independent mice.

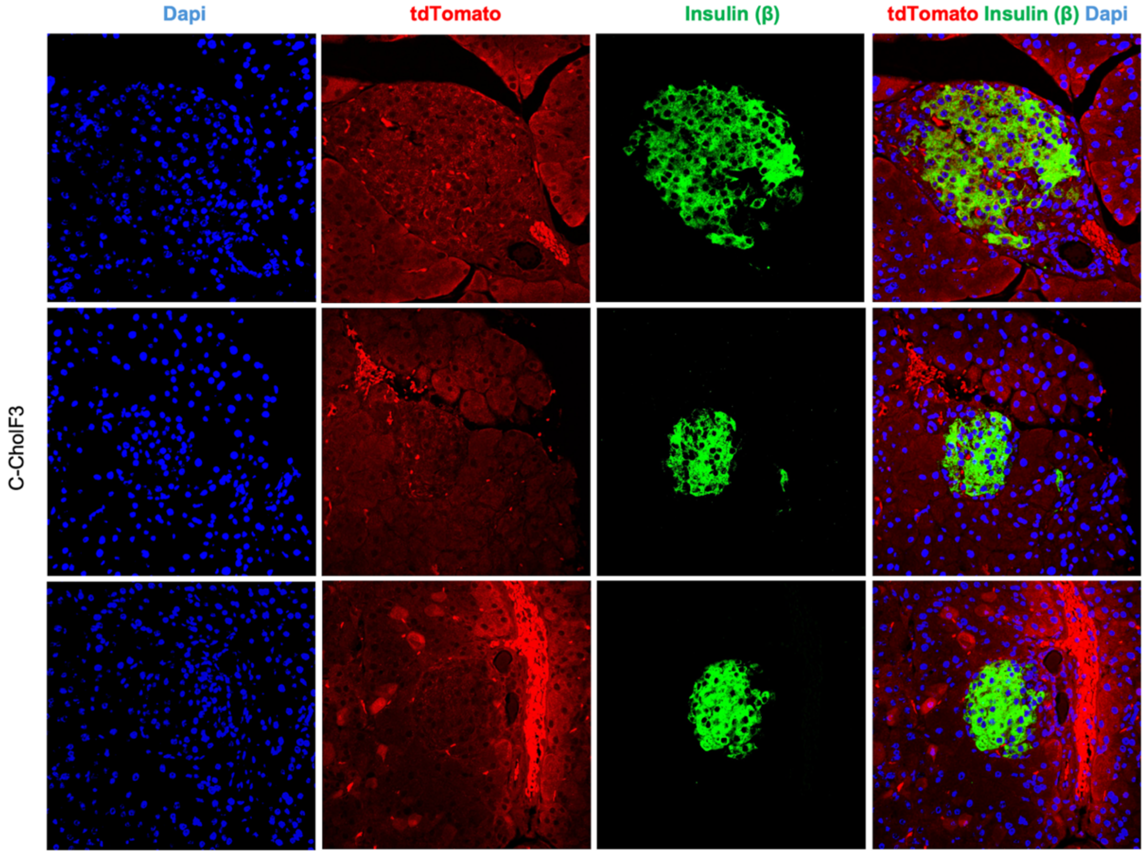

Figure S25. Replicates of images used to generate **Figure 7D**. Immunofluorescent images of pancreatic sections from C-CholF3 LNPs treated Ai14 mice at a dose of 1.5 mg/kg. Insulin antibody was used to stain the β-cells, and DAPI was used to label the nuclei. Images were captured at 40× magnification from three biologically independent mice.

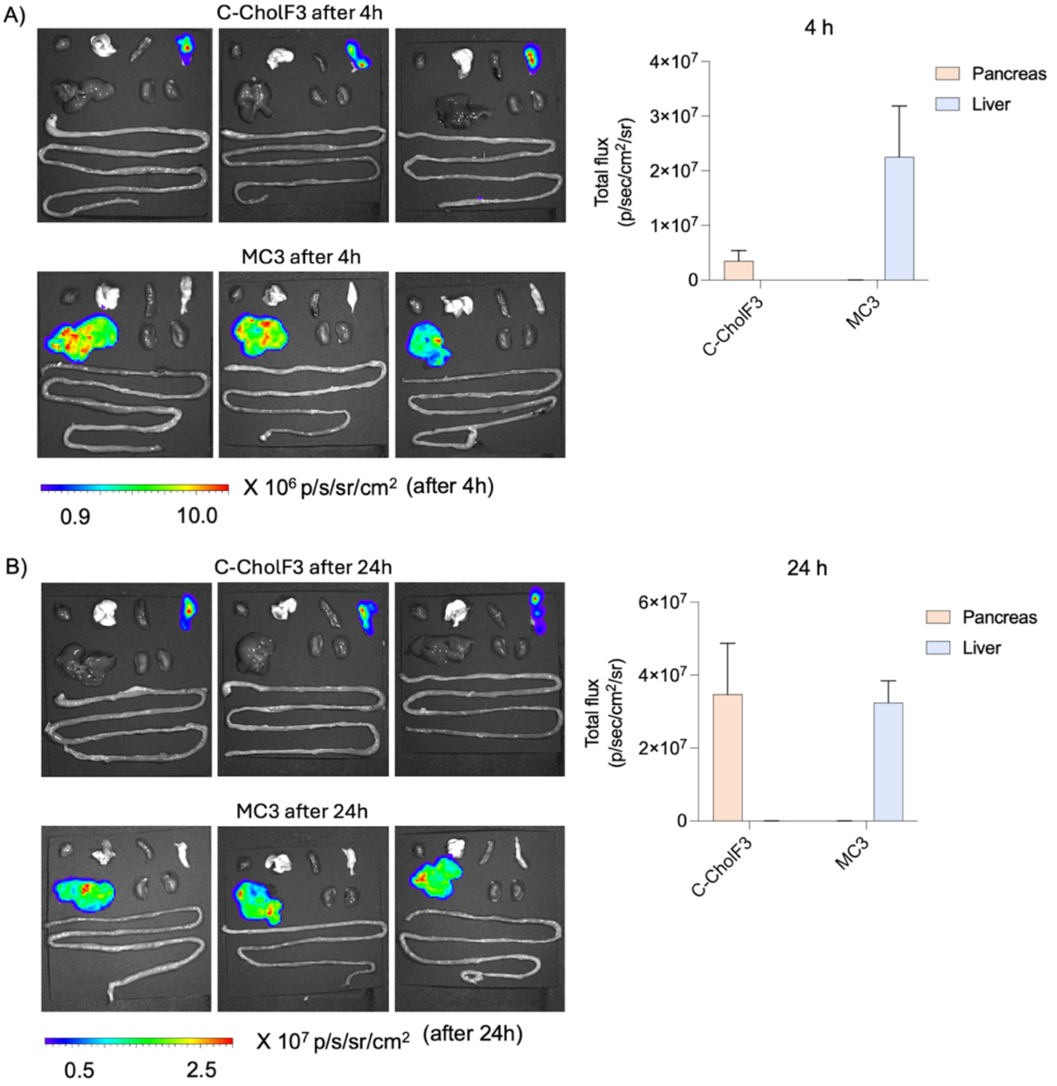

Figure S26. Comparison of protein expression of C-CholF3 ENDO LNPs with MC3 LNPs. A) Representative IVIS images at 4h post injection and graphical representation of total flux of C-CholF3 and MC3 mRNA LNPs injected intravenously at a dose of 0.5 mg/kg (n= 3 biologically independent mice) B) Representative IVIS images at 24h post injection and graphical representation of total flux of C-CholF3 and MC3 mRNA LNPs injected intravenously at a dose of 0.5 mg/kg (n= 3 biologically independent mice). Organs are arranged left to right as: heart, lung, spleen, pancreas, liver, kidneys and intestines.

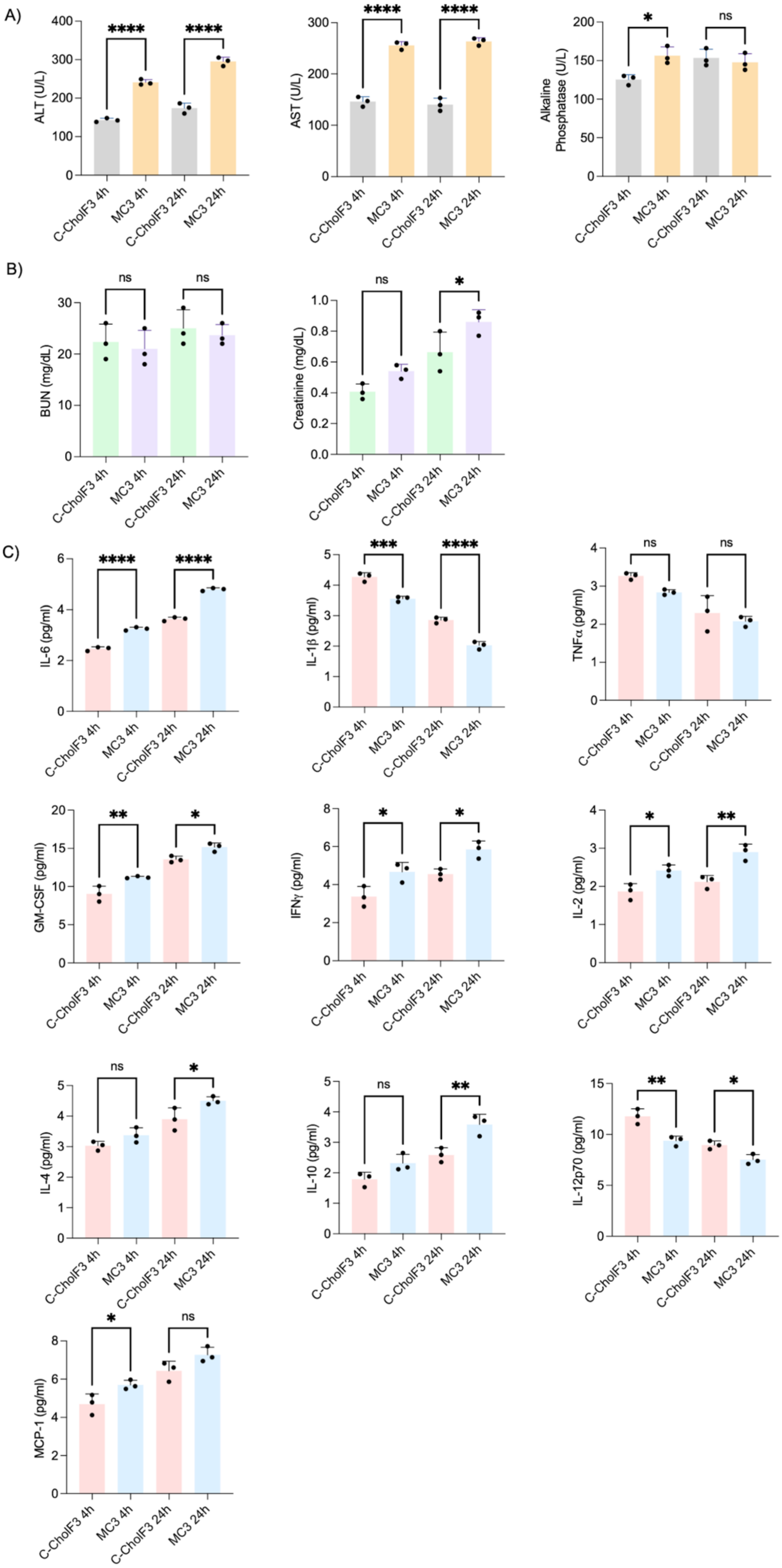

Figure S27. Comparison of toxicity and safety evaluation of C-CholF3 ENDO LNPs with MC3 LNPs. A) Serum levels of liver enzymes, alanine aminotransferase (ALT),

aspartate aminotransferase (AST) and Alkaline Phosphatase after intravenous administration with C-CholF3 and MC3 mRNA LNPs at 4 h and 24h (0.5 mg/kg, n = 3, ± SD, *P < 0.05, **P < 0.01, ***P < 0.001. NS, not significant, one-way ANOVA with Bonferroni post hoc analysis). B) Renal parameters, blood urea nitrogen (BUN), and creatinine after intravenous administration with C-CholF3 and MC3 mRNA LNPs at 4 h and 24h (0.5 mg/kg, n = 3, ± SD, *P < 0.05, **P < 0.01, ***P < 0.001. NS, not significant, one-way ANOVA with Bonferroni post hoc analysis). C) Levels of IL-6, IL-1β, TNFα, GM-CSF, IFNg, IL-2, IL-4, IL-10, IL-12p70 and MCP-1 in mice after intravenous administration with C-CholF3 and MC3 mRNA LNPs at 4 h and 24h (0.5 mg/kg, n = 3, ± SD, *P < 0.05, **P < 0.01, ***P < 0.001. NS, not significant, one-way ANOVA with Bonferroni post hoc analysis).

MATERIALS AND METHODS:

Materials

1,2-di-(9Z-octadecenoyl)-sn-glycero-3-phosphoethanolamine (DOPE), cholesterol and 1,2-dimyristoyl-rac-glycero-3-methoxypolyethylene glycol-2000 (DMG-PEG2K) were purchased from Avanti Polar Lipids, DLin-MC3-DMA, SM-102, 306Oi10, C12-200, Vitamin A, Vitamin D3, Vitamin K1, and (±)-α-Tocopherol was purchased from Cayman Chemicals. Riboflavin and Triton X-100 was purchased from Sigma. THP1 was synthesized in our lab using our established method.[1] Fetal Bovine serum was purchased from Gibco. QuantiT RiboGreen RNA Assay Kit was purchased from Invitrogen. FLuc mRNA N1-Me-Pseudo U (Catalog No. ON-279) and FLuc circRNA (Catalog No. ON-505) were purchased from Hongene Biotech (Union City, USA). CleanCap Cre mRNA (Catalog No. L-7211) was purchased from Trilink Biotechnology (San Diego, USA). Luciferase pcDNA (Plasmid #18964) was purchased from Addgene (Massachusetts, USA). The plasmid was further amplified and purified according to the manufacturer's protocol. HEK 293, BxPC-3 HUVEC and RAW 264.7 cell lines purchased from ATCC. HFF and HMC3 cell lines were given by Dr. Seungman Park's lab at UNLV. All the cells were cultured according to the ATCC guidelines. DMEM, RPMI and MEM growth medium (Gibco, USA) containing sodium bicarbonate, without sodium pyruvate and HEPES, was supplemented with 10% fetal bovine serum (Gibco, USA) and 1% penicillin/streptomycin (Thermo Fisher Scientific). Human umbilical vein endothelial cells were maintained in Ham's F12K medium (ATCC, USA) supplemented with 10% fetal bovine serum, 1% penicillin-streptomycin-amphotericin B (Fungizone) mix (BioWhittaker, Walkersville, Md.), 30 µg of endothelial cell growth supplement per ml, and 100 µg of heparin (Sigma) per ml. MeTC7 was purchased from MedChemExpress (New Jersey, USA). Recombinant Human Vitamin D Receptor was purchased from NovateinBio (Massachusetts, USA).

Formulation of Lipid Nanoparticles

LNPs were formulated using previously established protocol.[2] Briefly, the organic phase was prepared by dissolving DOPE and DMG-PEG200 in ethanol at a molar ratio of 16 and 2.5 respectively. The molar ratios of ionizable lipids, cholesterol and vitamins vary by formulation and range from 20 to 35% for ionizable lipids, 41.5 to 46.5% for Cholesterol and 5 to 15% for the fifth component. The generic four-component LNPs (SM-102, MC3, C12-200) which were used as controls were formulated in a molar ratio of 35:16:46.5:2.5. The aqueous phase was prepared by dissolving the corresponding mRNA in 10 mM citrate buffer at pH 3 (Teknova, Hollister, CA, USA). The ionizable lipid to mRNA weight ratio for all LNPs was 10:1. The hundred formulations were formulated in a 96-well plate, where each well had the ethanol phase and the aqueous phase was mixed rapidly in the well using a multichannel pipette. During validations, the two phases were loaded into separate glass syringes (Hamilton Company, Reno, NV) and LNPs were formed by chaotic mixing of the organic and aqueous phases at a 1:3 volume ratio in a microfluidic device using Fusion 400 X (Chemyx Inc, USA). The LNPs were subsequently dialyzed against 1X PBS (Thermo Fisher Scientific, Walthman, MA, USA) in 20 kDa molecular weight cutoff dialysis cassettes (Thermo Fisher Scientific) for 4 hours.

Characterization of Lipid Nanoparticles

The encapsulated mRNA concentration and encapsulation efficiency of the LNPs were assessed using the Quant-iT RiboGreen assay (Thermo Fisher Scientific), following established protocols.[3,4] Each LNP sample was diluted 100-fold in two microcentrifuge tubes, one containing 1X TE buffer and the other containing 1% (v/v) Triton X-100 (Sigma, USA) in 1X TE buffer. The Triton X-100 samples were mixed thoroughly and incubated for 5 minutes to lyse the LNPs. Samples (LNPs in 1X TE buffer, LNPs in 1% Triton X-100, and mRNA standards) were placed in quadruplicate in black-walled 96-well plates. The RiboGreen detection reagent was then added to each well according to the manufacturer's instructions. The plate was shaken at 200 rpm in the dark for 5 minutes, and fluorescence intensity was measured using a GloMax Explorer plate reader (Promega, USA) with an excitation wavelength of 480 nm and an emission wavelength of 520 nm. The encapsulated mRNA concentration was calculated from a standard curve generated using univariate least-squares linear regression. Encapsulation efficiency (EE) was determined using the formula: $1 - \frac{R_{TE}}{R_{TX}}$

where R_{TE} is the free RNA content in TE buffer, and R_{TX} is the total RNA content in 1% Triton X-100 buffer. The hydrodynamic diameter and polydispersity index (PDI) of the LNPs were measured using a Mobius instrument (Wyatt Technology, Santa Barbara, CA, USA). Each LNP sample was diluted 100-fold in 1X PBS and placed in a cuvette (Wyatt Technology) for analysis. The surface ζ-potential was also measured using the Mobius, with each LNP sample diluted 100-fold in deionized water (Thermo Fisher Scientific) and placed into a capillary cell for measurement. Size and ζ-potential were reported as mean ± standard deviation (n = 3 technical replicates).

TNS Assay

The pKa of LNPs was determined using a TNS (6-(p-Toluidino)-2-naphthalenesulfonic acid) binding assay. A stock solution of 0.16 mM TNS reagent (Sigma Aldrich) was prepared in deionized water. LNPs were diluted to a concentration of 40 ng/mL, and 10 µL of the TNS stock solution was added to each well. The final volume per well was adjusted to 250 µL, consisting of 150 mM sodium chloride, 20 mM sodium phosphate, 20 mM ammonium acetate, and 25 mM ammonium citrate. The assay was conducted across a pH range of 2 to 12, with increments of 0.5 pH units. Samples were placed in black 96-well plates and mixed on a plate shaker at 300 rpm for 5 minutes at room temperature in the dark. Fluorescence measurements were taken using a GloMax Explorer plate reader (Promega), with excitation and emission wavelengths set at 322 nm and 431 nm, respectively, each with a 20 nm bandwidth and a gain of 60. Normalized fluorescence data were plotted against pH, and the pKa value was determined as the pH corresponding to the inflection point of the titration curve.[5]

In Vitro Studies

Cells were seeded at a density of 18,000 cells per well in 100 µL of DMEM in a 96-well plate and allowed to adhere for 24 hours. After this, the media was removed, and 75 µL of fresh DMEM without penicillin-streptomycin was added. The cells were then treated with 125 ng of mRNA per 18,000 cells to evaluate *in vitro* luciferase expression mediated by each LNP. DMEM alone served as the negative control, while LNPs

formulated with MC3 were used as the positive control. The LNP-treated cells were incubated at 37°C for 24 hours. Following incubation, 100 µL of luciferase assay substrate (Promega) was added to each well. The plate was shaken on a plate reader at 200 rpm in the dark for 10 minutes, and luminescence intensity was measured using the GloMax Explorer plate reader (Promega, USA).

Cryo-TEM sample preparation and imaging

Three microliters of LNPs in a buffer solution were applied to a lacey copper grid coated with a continuous carbon film. The excess sample was carefully blotted away using the Gatan Cryo Plunge III. The grid was then mounted on a Gatan 626 single tilt cryo-holder and inserted into the TEM column. The specimen and holder tip were cooled with liquid nitrogen, maintaining the temperature throughout the transfer into the microscope and during imaging. Imaging was performed on a JEOL 2100 FEG microscope using the minimum dose method, essential for minimizing sample damage from the electron beam. The microscope was operated at 200 kV, with magnifications ranging from 10,000x to 60,000x to assess particle size and distribution. All images were recorded on a Gatan 2k x 2k UltraScan CCD camera.

Confocal Scanning Laser Microscopy (CLSM)

BxPC-3 cells were seeded in glass bottom dishes (Thermo Fisher, USA) and incubated for 24 h. The medium was then replaced with a culture medium containing DiO labelled LNPs (125 ng) for transfection for 2 hours. Briefly, LNPs encapsulating 125 ng of Fluc mRNA LNPs labelled with DiO (0.5% molar ratio of LNPs) were added to the dishes. Cell nuclei and lysosomes were stained with Hoescht 33342 (Lumiprobe, USA) and Lysotracker Red (Invitrogen, USA). Images were obtained using a confocal scanning laser microscope (Nikon A1, Japan) with a water immersion 63× objective lens. The imaging parameters were kept constant during the experiments.

H&E Staining

Hematoxylin and eosin (H&E) staining on pancreas and liver tissues from C57BL/6J mice were performed on formalin-fixed paraffin-embedded (FFPE) sections. Tissues were post-fixed in 10% formalin for 24–48 hours at room temperature (with the option to remain in formalin for up to a week) and then transferred directly to 70% ethanol. Histological processing was completed by HistoWiz Inc. (NY, USA) following their Standard Operating Procedure and fully automated workflow. Samples were embedded in paraffin and sectioned at 4 µm thickness. Formalin-fixed, paraffin-embedded tissue sections were deparaffinized in xylene, rehydrated through graded ethanol, and rinsed in distilled water. Sections were stained with Hematoxylin for 5 minutes, rinsed in tap water, differentiated in acid alcohol, and treated with a bluing reagent. Eosin staining was applied for 1–2 minutes, followed by brief rinsing. Slides were then dehydrated through graded ethanol, cleared in xylene, and coverslipped with a compatible mounting medium. Whole slide scanning at 40x magnification was performed using an Aperio AT2 system (Leica Biosystems).

Immunofluorescence

Unstained slides of Ai14 mouse pancreas tissue samples post-treatment were obtained from HistoWiz Inc. (NY, USA). Sections, 5 µm thick, were deparaffinized in xylene, rehydrated through a graded ethanol series, and subjected to antigen retrieval in citrate buffer (pH 6.0) at 95°C for 20 minutes. After cooling to room temperature, the sections were permeabilized with 0.1% Triton X-100 in PBS for 10 minutes and blocked with 5% normal goat serum in PBS for 1 hour. Immunofluorescent staining was performed using primary antibody against insulin (bio-techne, USA) followed by incubation with secondary antibody Alexa Fluor 488 (Thermo Fisher, USA) conjugated to fluorescent dyes. DAPI (1 µg/mL in PBS) was used for 10 minutes to stain nuclei. After washing with PBS, slides were mounted using an anti-fade mounting medium and covered with glass coverslips. Images were captured using a confocal scanning laser microscope (Nikon A1, Japan) with an oil immersion 40× objective lens with filters for tdTomato (red fluorescence), insulin (green fluorescence), and DAPI (blue fluorescence).

Multiplex Analysis of Cytokines

This study used Luminex xMAP technology for multiplexed quantification of 10 Mouse cytokines, chemokines and growth factors. The multiplexing analysis was performed using the Luminex™ 200 system (Luminex, Austin, TX, USA) by Eve Technologies Corp. (Calgary, Alberta). Ten markers were simultaneously measured in the samples using Eve Technologies' Mouse Focused 10-Plex Discovery Assay® (MilliporeSigma, Burlington, Massachusetts, USA) according to the manufacturer's protocol. The 10-plex consisted of GM-CSF, IFNγ, IL-1β, IL-2, IL-4, IL-6, IL-10, IL-12p70, MCP-1, and TNFα. Assay sensitivities of these markers range from 0.4 – 10.9 pg/mL for the 10-plex. Individual analyte sensitivity values are available in the MilliporeSigma MILLIPLEX® MAP protocol.

References

[1] I. Isaac, A. Shaikh, M. Bhatia, Q. Liu, S. Park, C. Bhattacharya, *ACS Nano* **2024**, *18*, 29045.

[2] K. J. Kauffman, J. R. Dorkin, J. H. Yang, M. W. Heartlein, F. DeRosa, F. F. Mir, O. S. Fenton, D. G. Anderson, *Nano Lett* **2015**, *15*, 7300.

[3] A.-G. Reinhart, A. Osterwald, P. Ringler, Y. Leiser, M. E. Lauer, R. E. Martin, C. Ullmer, F. Schumacher, C. Korn, M. Keller, *Mol. Pharmaceutics* **2023**, *20*, 6492.

[4] L. Cui, S. Pereira, S. Sonzini, S. van Pelt, S. M. Romanelli, L. Liang, D. Ulkoski, V. R. Krishnamurthy, E. Brannigan, C. Brankin, A. S. Desai, *Nanoscale* **2022**, *14*, 1480.

[5] M. J. Carrasco, S. Alishetty, M.-G. Alameh, H. Said, L. Wright, M. Paige, O. Soliman, D. Weissman, T. E. Cleveland, A. Grishaev, M. D. Buschmann, *Commun Biol* **2021**, *4*, 1.
